# MicroRNAs in Heart Failure Pathogenesis and Progression: Mechanistic Control, Biomarker Potential, and Translational Perspectives

**DOI:** 10.3390/life16030400

**Published:** 2026-03-01

**Authors:** Dorotea Zivalj, Lou Marie Salomé Schleicher, Antea Krsek, Hadid Joseph Farzad Diamee, Damir Raljevic, Lara Baticic

**Affiliations:** 1 Faculty of Medicine, University of Rijeka, 51000 Rijeka, Croatia; dzivalj@uniri.hr (D.Z.); loums.schleicher@uniri.hr (L.M.S.S.); antea.krsek@uniri.hr (A.K.); hadid.diamee@uniri.hr (H.J.F.D.); 2 Thalassotherapia Opatija, Clinic for Rehabilitation, Treatment and Prevention of Diseases of the Heart and Blood Vessels, Medical Faculty, University of Rijeka, 51410 Opatija, Croatia; damir.raljevic@tto.hr; 3 Department of Medical Chemistry, Biochemistry and Clinical Chemistry, Faculty of Medicine, University of Rijeka, 51000 Rijeka, Croatia

**Keywords:** biomarkers, cardiac remodeling, circulating microRNAs, heart failure, microRNAs, post-transcriptional regulation, therapeutic targeting

## Abstract

Heart failure (HF) remains a leading cause of morbidity and mortality worldwide and is driven by complex, interconnected pathophysiological processes, including maladaptive cardiac remodeling, fibrosis, hypertrophy, metabolic dysregulation, and cardiomyocyte loss. MicroRNAs (miRNAs), small non-coding RNAs that act as key post-transcriptional regulators of gene expression, have emerged as important coordinators of these processes across cardiomyocytes and non-myocyte cardiac cell populations. In addition to altered expression patterns, accumulating evidence indicates that miRNA activity is dynamically influenced by regulated biogenesis, maturation, and context-dependent mechanisms of action. Through reversible translational repression and longer-term mRNA destabilization, miRNAs support adaptive responses to acute cardiac stress, whereas their persistent dysregulation contributes to remodeling pathways that promote HF progression. This comprehensive narrative review provides an integrative overview of current knowledge on the role of miRNA networks in shaping the molecular heterogeneity of heart failure across disease stages, phenotypes, and cardiac cell types. Drawing on a broad body of experimental and clinical literature, we discuss advances in understanding miRNA biogenesis, post-transcriptional control, and cell-specific effects, while highlighting conceptual developments rather than applying systematic selection criteria. We further examine the translational and clinical implications of miRNA biology, critically considering the progress of miRNA-based therapeutics alongside the biological and practical challenges that continue to limit their widespread clinical implementation. In parallel, we explore the emerging potential of circulating miRNAs as minimally invasive biomarkers that reflect upstream regulatory stress at the level of RNA processing and post-transcriptional regulation. Finally, we address the growing application of artificial intelligence and machine learning approaches to high-dimensional miRNA datasets, which enable integrative analyses across clinical, imaging, and multi-omics domains and support biomarker discovery, patient stratification, and prediction of therapeutic response. Collectively, miRNA biology, supported by systems-level and AI-driven analytical frameworks, offers a unifying perspective for understanding, classifying, and monitoring cardiac remodeling in heart failure.

## 1. Introduction

### 1.1. Definition and Epidemiology of Heart Failure

Heart failure (HF) is consistently defined across the literature as a complex clinical syndrome arising from structural or functional cardiac abnormalities that impair ventricular filling or ejection. More specifically, HF represents the final common pathway of diverse etiologies characterized by impaired systolic and/or diastolic function, associated with high morbidity and mortality. The syndrome manifests clinically when reduced cardiac output results in insufficient perfusion of peripheral tissues [1]. Multiple sources distinguish HF subtypes based on ejection fraction. Heart failure with preserved ejection fraction (HFpEF) and heart failure with reduced ejection fraction (HFrEF) are recognized as distinct clinical entities [2,3]. The prognosis of HF patients remains poor. Among individuals aged 75 years and older, more than 8% are diagnosed with HF. In a 20-year real-world cohort study, the overall prevalence of HF was 2.1%, with a predominance in elderly patients [2,3,4]. Long-term cohort analyses have shown that five-year and ten-year mortality rates of HF are very high, highlighting the persistent burden of this condition [5]. Adverse prognostic indicators in heart failure include soluble suppression of tumorigenicity-2 (sST2) and Galectin-3 (Gal-3). Both biomarkers independently predict patient outcomes, with evidence showing that sST2 can either surpass or complement conventional markers in forecasting all-cause mortality among patients with acute decompensated HFrEF [6]. Consequently, these biomarkers are valuable tools for risk stratification and may inform clinical decisions in both chronic and acute HF management [7]. The epidemiological burden of heart failure continues to grow. In the United States, around 6.7 million adults currently live with HF, with a lifetime risk estimated at nearly 24% [8]. Globally, age-standardized HF prevalence is estimated to fall between 1–3% of the adult population [9]. Within the HF patient population, approximately 50% are believed to have HF with preserved ejection fraction (HFpEF), and temporal data suggest that the incidence of HFpEF has risen over time, while HFrEF has declined [10]. Age is a key risk factor: a large proportion of newly diagnosed HF patients are older adults with comorbid conditions such as hypertension and prior myocardial infarction [11]. The condition is associated with significant morbidity, frequent healthcare utilization, and early mortality. HF remains one of the leading causes of hospitalizations and is projected to impose increasing costs and resource demands in the coming decades [12]. Taken together, the growing epidemiological burden of heart failure and its persistently high morbidity and mortality underscore the need for a deeper understanding of the underlying biological mechanisms driving disease onset and progression. In this context, heart failure is increasingly recognized not merely as a hemodynamic disorder, but as the result of complex and interconnected pathophysiological processes involving myocardial remodeling, fibrosis, hypertrophy, and cardiomyocyte death, which are discussed in detail in the following section.

### 1.2. Major Pathophysiological Mechanisms Driving Heart Failure: Myocardial Remodeling, Fibrosis, Hypertrophy, and Cell Death

Myocardial remodeling in heart failure represents a critical and multifaceted pathophysiological process, involving cardiomyocyte hypertrophy, extracellular matrix (ECM) expansion, and inflammatory cell death, which together drive structural, functional, and molecular changes in the heart [13]. Distinguishing causal miRNAs from associative biomarkers remains a key challenge in heart failure research. Causality can be strengthened through complementary approaches, including genetic analyses such as Mendelian randomization, functional perturbation using targeted editing or inhibition, and careful validation of downstream gene targets. Agreement between myocardial and circulating miRNA levels supports biological relevance, while longitudinal studies help determine whether changes precede disease progression. Dysregulated miRNA networks are likely not only biomarkers but active contributors to the distinct mechanisms underlying different heart failure phenotypes. In HFpEF, altered miRNAs are closely linked to pathways involved in inflammation, endothelial dysfunction, and impaired metabolism, reflecting the systemic inflammatory and metabolic disturbances that define this condition. In contrast, HFrEF is more strongly associated with miRNAs that regulate cardiomyocyte death and fibrotic remodeling, processes central to ventricular dilation and contractile dysfunction. Developing these mechanistic links more explicitly would strengthen the discussion beyond descriptive biomarker profiling.

In response to chronic stress, such as pressure overload or neurohormonal activation, cardiomyocytes initially undergo adaptive hypertrophy, but prolonged overload leads to maladaptive growth and contractile dysfunction [14]. At the same time, cardiac fibroblasts become activated and deposit excessive collagen (notably collagen I/III), resulting in interstitial fibrosis and increased myocardial stiffness [15]. Concomitantly, programmed cell death (PCD) via pathways such as pyroptosis, mediated by NLRP3 inflammasome activation and caspase-1-dependent cleavage of gasdermin D, plays a critical role in remodeling by promoting cardiomyocyte loss and inflammation [16]. Autophagy also contributes in a complex way: while basal autophagic flux helps remove damaged organelles and maintain cardiomyocyte viability, its dysregulation can worsen remodeling by promoting cell death [17]. Moreover, metabolic disturbances and oxidative stress amplify the remodeling process by stimulating inflammatory and fibrotic signaling [18]. From a therapeutic perspective, sodium-glucose co-transporter 2 inhibitors (SGLT2i) have shown antifibrotic and anti-inflammatory effects in models of HF [19] and reduced cardiac inflammation and fibroblast activation in animal models independent of SGLT2 expression. Cardiac fibrosis is a central feature of pathological myocardial remodeling and is defined by the excessive accumulation of extracellular matrix (ECM), predominantly collagen types I and III, which progressively impairs tissue elasticity and LV compliance. These structural alterations contribute directly to both systolic and diastolic dysfunction [20]. Two predominant patterns are recognized: replacement fibrosis, occurring after cardiomyocyte death, and interstitial (reactive) fibrosis, which arises from chronic neurohormonal activation or mechanical overload and is strongly associated with HFpEF. Reactive fibrosis is diffusely distributed and reflects chronic pressure or metabolic stress rather than acute injury [21].

Cardiac fibrosis is mainly driven by resident fibroblasts, which differentiate into extracellular matrix-producing myofibroblasts. Other cells, such as endothelial cells through EndoMT, pericytes, and inflammatory cells, can also contribute. Single-cell studies show that fibroblasts consist of different subpopulations with distinct inflammatory and matrix-producing profiles [22]. The TGF β and SMAD pathway is the central driver of fibroblast activation and collagen synthesis and often remains active after the initial injury [23]. It interacts with RAAS signaling, where angiotensin II promotes oxidative stress and matrix deposition through the AT1 receptor. Additional pathways, including Wnt and β catenin, Endothelin 1, and Notch, further enhance fibroblast activation, especially during pressure overload [24]. Chronic inflammation, altered MMP and TIMP balance, and mechanical stretch through integrin FAK and Src signaling also promote fibrosis [25,26]. Metabolic changes and reactive oxygen species, together with YAP and TAZ signaling, further support fibroblast activation [27].

HFpEF is commonly associated with systemic inflammation, endothelial dysfunction, obesity, and diabetes, leading to diffuse interstitial fibrosis and increased myocardial stiffness [28]. In contrast, HFrEF more often shows focal replacement fibrosis due to cardiomyocyte loss after ischemic injury [29]. Cardiac magnetic resonance with T1 mapping and extracellular volume measurement allows noninvasive assessment of interstitial fibrosis and has prognostic value [30]. Circulating markers such as Galectin 3 reflect inflammation and fibrosis and provide prognostic information, while procollagen peptides such as PICP and PIIINP are still under evaluation [31,32].

Cardiac hypertrophy is an adaptive response to increased hemodynamic load and involves enlarged cardiomyocytes, fetal gene reactivation, metabolic changes, and altered calcium handling [33]. Physiological hypertrophy, such as with exercise, is reversible, whereas pathological hypertrophy is linked to fibrosis and heart failure [34,35]. Genetic forms such as hypertrophic and dilated cardiomyopathy arise from mutations in sarcomeric or cytoskeletal proteins that disturb mechanotransduction and calcium signaling [36]. Calcium-dependent pathways activate calcineurin, NFAT, and CaMKII, which promote hypertrophic gene expression [37]. Transcription factors such as MEF2 and GATA4 are tightly regulated by HDACs and epigenetic modifiers including p300 and histone methylation enzymes [38,39,40,41]. Noncoding RNAs also modulate hypertrophic signaling and stress responses [42]. Persistent stress from hypertension, valve disease, myocardial infarction, or genetic mutations drives maladaptive remodeling with fibrosis and contractile dysfunction [43,44].

Loss of cardiomyocytes through regulated cell death is another key mechanism in heart failure [45]. Besides apoptosis and necrosis, pyroptosis and ferroptosis also contribute [46,47]. Oxidative stress activates ASK1 and promotes both apoptotic and necrotic pathways [48]. Reactive oxygen species can trigger inflammasome activation, leading to pyroptosis and release of IL 1β and IL 18, which worsen inflammation and remodeling [49]. Ferroptosis, driven by iron-dependent lipid peroxidation and GPX4 inactivation, also contributes to cardiomyocyte loss [50]. Interactions between ferroptosis and pyroptosis have been observed, for example through NLRP3 signaling in metabolic stress [51]. Together, these cell death pathways reduce contractile mass and promote fibrosis and dysfunction [52]. In experimental models, inhibition of ASK1 reduces fibrosis and hypertrophy by limiting p38 MAPK signaling [53].

### 1.3. MicroRNAs in Heart Failure: Structure, Biogenesis, and Regulatory Function

**Figure 1:**
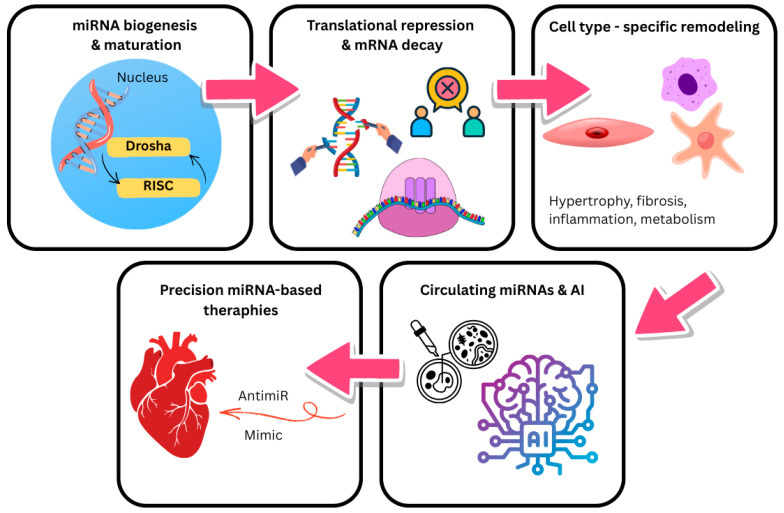
MicroRNA Pathways in Cardiac Remodeling and Precision Therapeutics.

MicroRNAs (miRNAs) are short, single-stranded, non-coding RNAs that regulate gene expression post-transcriptionally by guiding the RNA-induced silencing complex (RISC) to complementary sites on target mRNAs, thereby causing translational repression or mRNA decay [54]. The canonical biogenesis pathway begins with transcription of primary miRNA transcripts (pri-miRNAs), usually by RNA polymerase II, followed by nuclear cropping by the Drosha–DGCR8 complex into precursor hairpins (pre-miRNAs). Pre-miRNAs are exported to the cytoplasm by Exportin-5/Ran-GTP and are subsequently cleaved by Dicer to produce a mature miRNA duplex; one strand is loaded into RISC while the passenger strand is degraded or sometimes retained as a functional miRNA [55]. Recent mechanistic and decay-pathway studies have refined this model and documented regulatory checkpoints that determine miRNA stability and turnover [56]. Functionally, individual miRNAs can target dozens to hundreds of mRNAs, allowing coordinated regulation of entire pathways relevant to cardiac biology, including apoptosis, hypertrophy, fibrosis, inflammation, angiogenesis, and metabolic remodeling [57]. In the heart, tissue-restricted and stress-responsive miRNAs have been shown to modulate cardiomyocyte survival, fibroblast activation, endothelial function, and electrophysiological properties, linking miRNA dysregulation to the molecular mechanisms that drive heart failure [58]. Many miRNAs are detectable and extremely persistent in biofluids (plasma/serum, exosomes), where disease-specific expression patterns correspond with the existence, etiology, and prognosis of heart failure, which is significant for translational applications. The potential and present difficulties (preanalytical variability, normalization, and cohort heterogeneity) of circulating miRNAs as diagnostic and prognostic biomarkers in HF have been highlighted by several extensive profiling studies and meta-analyses [59]. Lastly, significant preclinical progress and early clinical interest in miRNA-based therapeutics for cardiovascular disease have been fueled by the ability to either replace protective miRNAs (miRNA mimics) or inhibit pathogenic miRNAs (antagomirs/anti-miRs). However, safety, tissue specificity, and durable delivery are still active areas of research [60]. Therefore, this review aims to comprehensively summarize the current understanding of the role of microRNAs in the pathophysiology and progression of heart failure, with an emphasis on their potential as novel therapeutic targets and their diagnostic and prognostic utility as circulating biomarkers (Figure 1).

## 2. Biogenesis and Mechanisms of microRNA Action

### 2.1. Transcription and Processing of Primary miRNA (Pri-miRNA → Pre-miRNA → Mature miRNA)

Most microRNA genes are transcribed by RNA polymerase II into long primary transcripts called pri miRNAs, which form stem loop structures. In the nucleus, the microprocessor complex, composed of Drosha and DGCR8, cleaves pri miRNAs into about 70 nucleotide precursor miRNAs (Figure 1). This first step is essential for normal miRNA biogenesis. The precursor miRNA is then exported to the cytoplasm by Exportin 5 and Ran GTP for further processing [61].

In heart failure, cardiomyocytes and other cardiac cells are exposed to chronic mechanical stress, oxidative damage, and inflammation. Disturbances at this early processing step may therefore strongly affect the availability of stress-responsive and cell-type-specific miRNAs that regulate remodeling. MiRNA genes are tightly controlled by promoters and transcription factors, linking their expression to signaling pathways and developmental programs [62].

Alternative export pathways may also support miRNA maturation under certain conditions. For example, during cellular quiescence, Exportin 1 can partly replace Exportin 5, allowing some capped pri miRNAs to be exported and processed in the cytoplasm [63]. Although mainly studied outside the cardiovascular system, these mechanisms show that miRNA biogenesis is flexible and may adapt in heart failure when normal nuclear export is impaired. In addition, structural features of precursor miRNAs, such as the 3′ overhang and apical loop, can influence Exportin 5 binding and export efficiency [64].

Microprocessor activity is further regulated by RNA binding proteins such as DDX5, DDX17, and SMADs, and by specific sequence motifs that enhance Drosha and DGCR8 processing [65]. This link between signaling pathways and miRNA processing is highly relevant in heart failure, where TGF β and SMAD signaling, inflammation, and mechanical stress shape fibroblast activation and cardiomyocyte hypertrophy [23].

Overall, miRNA biogenesis is not a simple linear pathway but a regulated process with several checkpoints [66]. Disruption at early stages, whether due to impaired processing or export, may amplify downstream gene network disturbances involved in fibrosis, hypertrophy, metabolic remodeling, and cell survival in heart failure.

**Figure 2:**
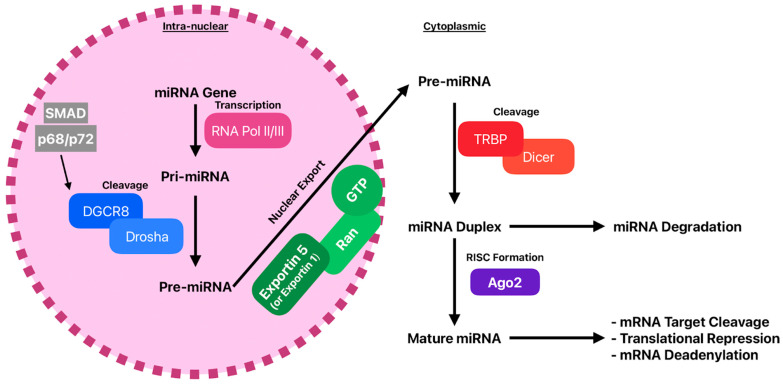
Schematic Overview of Nuclear and Cytoplasmic Processing of miRNAs and their Role in Post-transcriptional Gene Regulation.

After nuclear export, precursor miRNAs are processed in the cytoplasm by Dicer and loaded into the RNA-induced silencing complex, which are key steps in miRNA maturation (Figure 2). Dicer cleaves the hairpin structure into a 22-nucleotide miRNA duplex and is regulated by associated proteins such as TRBP, PACT, and ADAR, which influence cleavage accuracy and loading into the complex [67]. Dicer activity is also modulated by post-translational modifications such as phosphorylation, linking miRNA processing to cellular stress and signaling pathways [68]. In addition to canonical miRNA maturation, Dicer participates in noncanonical pathways, including the processing of mirtrons [69].

In heart failure, cardiomyocytes and fibroblasts are exposed to oxidative stress, inflammation, and metabolic imbalance. Changes in Dicer activity or complex assembly may therefore alter the availability and function of miRNAs involved in hypertrophy, fibrosis, and cell survival. After Dicer cleavage, one strand of the duplex, the guide strand, is loaded into an Argonaute protein, while the passenger strand is degraded. Strand selection depends on sequence features and thermodynamic stability, especially within the seed region at nucleotides 2 to 8, which determines target recognition [54].

Argonaute proteins are central components of the silencing complex and mediate gene repression by inhibiting translation or, in the case of AGO2 and sometimes AGO3, by cleaving target mRNAs [54,70,71]. Their conserved domains anchor the guide RNA and enable catalytic activity [72]. Argonaute function is regulated by post-translational modifications and interactions with partner proteins such as the TNRC6 family, which influence target binding and silencing efficiency [70]. In some cell types, Argonaute proteins are also found in the nucleus, where they contribute to transcriptional and post-transcriptional regulation [73].

Additional regulatory layers at the level of microprocessor activity, cofactor interactions, nuclear organization, and noncanonical Drosha functions further shape mature miRNA abundance and strand selection [74,75,76,77,78,79]. Together, these checkpoints show that Dicer processing and silencing complex assembly are dynamic control points. In heart failure, stress-induced alterations at this level may reprogram gene regulatory networks and drive maladaptive remodeling.

MicroRNAs regulate gene expression after transcription by controlling translation and mRNA stability. Mature miRNAs are loaded into the RNA-induced silencing complex, whose core component is an Argonaute protein. Guided by sequence complementarity, the complex binds mainly to the 3′ untranslated region of target mRNAs and induces translational repression or mRNA degradation, which are closely linked processes [61].

In many cases, regulation starts with translational repression. The miRNA complex can block cap-dependent initiation by interfering with eIF4E and eIF4G interactions or by recruiting repressors such as DDX6 and 4E T, thereby preventing formation of the pre-initiation complex [80]. Experimental studies show that miRNAs can reduce protein synthesis without immediate mRNA decay, as target transcripts shift to lighter polysomes rather than being rapidly degraded [81]. This mechanism is flexible and reversible, allowing rapid adaptation to changing cellular needs [82]. In vivo inhibition of miRNA function leads to quick restoration of target gene expression, supporting this dynamic model [83].

In the heart, where cells face constant hemodynamic, metabolic, and ischemic stress, reversible translational repression allows rapid adjustment of protein output without changing mRNA levels [82]. During acute stress, miRNAs can temporarily suppress transcripts involved in hypertrophy, calcium handling, or metabolism, providing short-term protection [84]. With persistent stress, repression often shifts toward mRNA destabilization, reinforcing maladaptive pathways linked to fibrosis, metabolic imbalance, and contractile dysfunction [85,86]. This reflects a transition from adaptive fine tuning to sustained gene silencing.

Mechanistically, prolonged binding of the miRNA complex recruits GW182 and TNRC6 proteins, which connect Argonaute to the CCR4 NOT deadenylase complex and decapping machinery [87]. Shortening of the poly A tail promotes transcript decay, and genome-wide analyses indicate that mRNA destabilization accounts for much of miRNA-mediated regulation at steady state [82,88]. The final outcome depends on factors such as miRNA and target abundance, binding site number, RNA binding proteins, mRNA half life, and cellular context [89]. Codon usage and ribosome dynamics can also influence stability [90]. In addition, target-directed miRNA degradation can reduce specific miRNA levels through the ZSWIM8 pathway, adding further control [91].

In heart failure, this dual mode of action is highly relevant. miRNAs may first act through reversible translational repression during early or compensatory stages, but with ongoing stress they increasingly drive mRNA decay and stabilize maladaptive remodeling programs [92]. Defining how this balance changes across disease stages and phenotypes may improve molecular classification of heart failure [93]. It is also important to distinguish disease stage, remodeling type, and ejection fraction phenotype, since these represent overlapping but distinct aspects of heart failure biology. Together, these mechanisms explain how miRNAs shape cardiomyocyte function and cardiac remodeling.

### 2.2. Functional Importance of miRNAs in Cardiomyocytes and Cardiac Tissue Homeostasis

Within this mechanistic framework, disease-associated microRNAs in heart failure emerge not merely as passive markers of transcriptional change, but as active regulators whose effects depend on the dynamic balance between translational repression and mRNA decay across specific cardiac cell types and disease stages. MicroRNAs are indispensable regulators of gene expression in cardiomyocytes and throughout the cardiac microenvironment. By modulating the translation and stability of large mRNA networks, they coordinate contractile performance, excitation–contraction coupling, mitochondrial bioenergetics, cell survival, and intercellular communication [94]. Among cardiomyocyte-enriched miRNAs, miR-1, miR-133a, and miR-208a are particularly critical. miR-1 regulates ion channel gene expression, conduction pathways, and electrophysiological stability, and its dysregulation predisposes to arrhythmias and impaired conduction [95]. miR-133a suppresses pathological hypertrophy and limits apoptosis; its loss increases susceptibility to hypertrophic growth and cardiomyocyte death [96]. Meanwhile, miR-208a is instrumental in controlling myosin heavy chain isoform switching and contractile kinetics, thereby regulating adaptive responses in the stressed heart [97]. Beyond cardiomyocytes, miRNAs maintain tissue-level homeostasis by regulating fibroblasts, endothelial cells, and immune cells within the cardiac microenvironment. In cardiac fibroblasts, downregulation of miR-29 promotes collagen synthesis and extracellular matrix accumulation, thereby driving fibrotic remodeling and increased myocardial stiffness [98]. Endothelial and immune cell-derived miRNAs further shape vascular integrity, inflammatory tone, and intercellular signaling, highlighting the multicellular nature of miRNA-mediated regulation in the failing heart. MicroRNAs also play a central role in regulating mitochondrial and metabolic programs that sustain the high energetic demands of cardiac tissue. During the fetal-to-postnatal transition, coordinated miRNA–mRNA networks drive the metabolic switch toward fatty acid oxidation and oxidative phosphorylation. In adult cardiomyocytes, miRNAs continue to fine-tune substrate utilization, reactive oxygen species detoxification, and mitophagy. Dysregulation of these networks during ischemia, pressure overload, or hypertrophic stress disrupts mitochondrial function, leading to energetic insufficiency, oxidative damage, and impaired contractile performance [99]. Through flexible and context-dependent control of protein output, miRNAs enable the myocardium to respond rapidly to acute stress via reversible translational repression, while under sustained pathological load, they facilitate longer-term remodeling through mRNA destabilization and transcript decay. When these regulatory programs become chronically dysregulated, the resulting failure to restore cellular and tissue homeostasis contributes to hypertrophy, arrhythmogenesis, fibrosis, metabolic inflexibility, and progressive heart failure. Accordingly, identifying specific miRNAs, their dominant targets, and the molecular nodes they control remains a major research priority with significant therapeutic implications [92,100]. While these mechanistic layers collectively define how miRNAs shape cardiomyocyte behavior and broader cardiac tissue homeostasis, their clinical translation remains uneven. This gap underscores the need for a critical appraisal of which insights from miRNA biology are most likely to yield actionable advances in heart failure diagnosis, risk stratification, and treatment.

### 2.3. Translational and Clinical Implications of miRNA Biogenesis

Building on the central role of microRNAs in coordinating cardiac gene regulation, advances in miRNA biogenesis provide a mechanistic framework for understanding how molecular perturbations are translated into phenotypic diversity in heart failure.

MicroRNA biogenesis and maturation, encompassing Drosha–DGCR8 processing, Exportin-5-mediated nuclear export, Dicer cleavage, and RISC loading, are now characterized with high molecular resolution [61,65,67,72,74]. This depth of mechanistic understanding has been instrumental in defining how perturbations in miRNA abundance, processing fidelity, or strand selection can reshape cardiac signaling networks involved in hypertrophy, fibrosis, inflammation, and cell death [22,54,92,100]. However, from a translational perspective, the field has reached a stage where further refinement of biogenetic mechanisms is unlikely to yield proportional translational benefit unless directly linked to human disease phenotypes or therapeutic feasibility [101,102].

Despite extensive molecular insight, the translational penetration of miRNA biology into heart failure management remains limited [101,102,103]. Most studies interrogating miRNA biogenesis, stability, strand selection, and RISC dynamics are conducted in vitro or in small-animal models. While these systems are indispensable for establishing causal relationships, their ability to predict therapeutic efficacy or long-term safety in the heterogeneous, comorbidity-laden heart failure population is inherently constrained [22,104,105]. Notably, relatively few investigations incorporate longitudinal human samples, treatment-response data, or hard clinical endpoints, which limit their immediate clinical relevance [106,107,108,109].

Across the broader RNA research landscape in heart failure, a clear imbalance persists between discovery and intervention. Most studies remain descriptive or mechanistic, focusing on cataloguing dysregulated miRNAs, targets, or pathways, whereas only a small fraction advance toward therapeutic testing. To date, anti-miR-132 (CDR132L) represents the only miRNA-directed therapy to progress into controlled human clinical trials in heart failure, underscoring both the translational promise of miRNA-based approaches and the current bottleneck of the field [110,111,112,113,114]. This singular example illustrates that clinical translation is achievable, but only under conditions of rigorous target selection, robust preclinical validation in large-animal models, and clearly defined disease contexts [110,112,114]. Importantly, several miRNAs with robust and reproducible effects in preclinical models have shown limited or divergent relevance in human heart failure, underscoring key translational discrepancies between experimental systems and clinical disease. To illustrate recurrent barriers to clinical translation, we highlight three well-characterized miRNAs selected as representative archetypes (Table 1). These examples reflect common patterns observed across the field, including divergence between biomarker and therapeutic relevance, limited reproducibility of mechanistic findings in human cohorts, and species-dependent regulatory effects.

Several biological and practical barriers explain why detailed knowledge of miRNA biogenesis has not yet translated into broad clinical use. miRNA processing is highly cell-type-specific and varies between cardiomyocytes, fibroblasts, endothelial cells, and immune cells. Individual miRNAs also regulate many genes, raising concerns about off-target effects and long-term safety when used therapeutically. In addition, challenges in cardiac-specific delivery, dose control, and reversibility still limit clinical application beyond early experimental studies [102,120,121,122].

In the near term, the greatest clinical value of miRNA research may lie in biomarker-based precision phenotyping rather than direct therapeutic modulation. Circulating miRNAs can reflect early molecular remodeling before clear functional decline and may capture disease processes not fully detected by traditional biomarkers. When combined with imaging, proteomics, and clinical data, they may improve early diagnosis, risk stratification, and therapy monitoring, especially in conditions such as HFpEF where current tools are limited [10,11,108,123].

Over the next decade, a realistic goal is to integrate validated multi-miRNA signatures into clinical decision-making rather than to widely use miRNA-based drugs [108,124,125,126,127]. Although some selected therapies may reach advanced trials, broader use will require better delivery systems, safety evaluation, and patient selection strategies [102,120,121,122]. Thus, the detailed understanding of miRNA biology should serve as a foundation for integrative and clinically focused research [101,102,128,129].

## 3. Deregulation of microRNA Expression in Heart Failure

### 3.1. Altered miRNA Expression During Cardiac Remodeling

In response to pathological stressors such as pressure overload, myocardial infarction, and persistent neurohormonal activation, the heart undergoes a complex process known as cardiac remodeling. Dysregulation of microRNA (miRNA) expression is a hallmark of this remodeling and contributes to structural, electrical, and metabolic alterations in the heart [130]. MiRNAs regulate post-transcriptional gene expression in both cardiomyocytes and non-myocyte cardiac cells, orchestrating the heart’s adaptive and maladaptive responses [115]. Altered miRNA expression patterns are now well documented in human failing hearts and experimental models, reflecting dynamic myocardial adaptation to stress and the eventual breakdown of compensatory mechanisms. Certain miRNAs are consistently upregulated during remodeling and drive maladaptive responses. For instance, miR-34a promotes cardiomyocyte apoptosis, reduces angiogenesis, and increases fibrosis in post-infarction and pressure-overload models. Therapeutic inhibition of the miR-34 family attenuates pathological remodeling and improves cardiac function [131]. Similarly, miR-21 is elevated in stressed hearts, promoting fibroblast activation, pro-fibrotic signaling, and extracellular matrix deposition, thereby contributing to structural remodeling [115]. Conversely, some miRNAs are downregulated, releasing constraints on hypertrophy, fibrosis, and cell death. For example, miR-133 levels are decreased in failing hearts, and its suppression is associated with heightened risk of hypertrophy, fibrosis, and arrhythmia [132]. Importantly, miRNA expression changes are context-dependent, varying with the type of cardiac stress, cell population, and disease stage. For instance, miR-148a is upregulated in pressure-overload (concentric) remodeling but downregulated in dilated cardiomyopathy (eccentric remodeling), reflecting distinct miRNA programs linked to remodeling phenotypes [133]. Cell-type-specific miRNA signatures further contribute to remodeling heterogeneity. Cardiac fibroblasts, endothelial cells, and inflammatory cells modify miRNA output differently than cardiomyocytes, affecting extracellular matrix regulation, paracrine signaling, and intercellular communication [134]. Cardiomyocyte size and survival, electrophysiological behavior, fibroblast activation and behavior, vascular and endothelial responses, and extracellular matrix regulation are all impacted by the altered miRNA landscape during cardiac remodeling [135]. The altered miRNA landscape in cardiac remodeling influences cardiomyocyte size and survival, electrophysiological behavior, fibroblast activation, vascular and endothelial responses, and extracellular matrix organization [136].

### 3.2. microRNAs Involved in Cardiomyocyte Hypertrophy (e.g., miR-21, miR-133, miR-1)

Cardiomyocyte hypertrophy is a key feature of pathological remodeling and is characterized by increased cell size, enhanced protein synthesis, and re-expression of fetal genes such as atrial natriuretic peptide and β-myosin heavy chain [135]. It is driven by altered calcium handling, calcineurin and NFAT activation, sarcoplasmic reticulum dysfunction, and contractile protein changes [137]. miRNAs coordinate these processes by regulating mRNAs involved in growth, apoptosis, structural integrity, and ion channels [138]. For example, miR-133 and miR-1 counteract hypertrophy by inhibiting RhoA-, Cdc42-, and Ca^2+^-dependent signaling, whereas other miRNAs enhance ERK or PI3K/AKT pathways and promote metabolic shifts [139]. Dysregulation of this miRNA network contributes to maladaptive hypertrophy and heart failure progression [135].

MiR-21 is frequently upregulated in hypertrophy and failing hearts and acts as a key paracrine mediator between fibroblasts and cardiomyocytes. Fibroblast-derived exosomal miR-21, particularly miR-21-3p, promotes cardiomyocyte hypertrophy by targeting SORBS2 and PDLIM5, which regulate cytoskeletal organization [140]. miR-21 also enhances AKT, ERK, and SMAD signaling by suppressing PTEN and modulating TGF-β pathways, thereby promoting growth and fibrosis [141,142]. Inhibition of miR-21 reduces hypertrophy and adverse remodeling in experimental models, supporting its therapeutic relevance [143,144].

In contrast, miR-133a is a muscle-specific miRNA that suppresses pathological hypertrophy and fibrosis. It is consistently downregulated in pressure overload, ischemia, and diabetic cardiomyopathy, while its restoration reduces hypertrophy and fibrosis and improves function [145,146]. In diabetic Akita mice, miR-133a overexpression attenuates fibrosis, limits β-myosin heavy chain induction, and preserves SERCA2a levels, linking it to calcium handling and contractile control [147]. Its activity is further regulated by m6A-dependent mechanisms involving IGF2BP2 and FTO, which enhance repression of pro-hypertrophic targets such as CDC42 and CTGF [148]. Stem cell and progenitor cell studies confirm that miR-133a reduces fibrosis and apoptosis and improves cardiac repair [149,150,151], partly by limiting TGF-β/SMAD signaling and connective tissue growth factor expression [132]. These findings identify miR-133a as a central brake on maladaptive remodeling.

Similarly, miR-1 is often downregulated in pathological hypertrophy. Its restoration reverses hypertrophy, reduces fibrosis and apoptosis, and improves calcium handling in pressure overload models [152]. miR-1 directly targets IGF-1 and IGF-1R, as well as cytoskeletal regulators such as TWF1, thereby restraining growth signaling and structural remodeling [135,153]. It also modulates calcium-dependent transcription through calmodulin, NFAT, and MEF2, and may influence mitochondrial energetics via IGF-1-related calcium signaling [140,154]. Increased miR-1 during endurance training suggests a role in adaptive remodeling as well [155]. Together, miR-1, miR-133a, and miR-21 form key regulatory nodes that shape the balance between adaptive and maladaptive hypertrophic remodeling.

### 3.3. Regulatory Networks and Additional miRNAs

The progression and magnitude of the hypertrophic response are determined by the interaction of pro-growth and anti-growth signals within a complex regulatory network mediated by these miRNAs. Under pathological conditions, miR-26a-5p displays pro-hypertrophic properties. Its expression is upregulated and induces autophagy in phenylephrine-stimulated cardiomyocytes. This autophagic response subsequently activates the NLRP3 inflammasome through increased expression of NLRP3, ASC, and Caspase-1. Overexpression of miR-26a-5p also promotes cardiomyocyte hypertrophy and interstitial fibrosis in a rat model of pressure overload (TAC), corroborating in vitro findings [156]. Furthermore, miR-26a-5p has been shown to target ADAM17. Restoration of miR-26a-5p expression attenuates hypertrophic responses by suppressing ADAM17 [157]. These findings indicate a dual role for miR-26a-5p in modulating proteolytic and inflammatory mechanisms that contribute to pathological remodeling. miR-222 exhibits context-dependent effects. Initially associated with physiological hypertrophy in response to exercise, miR-222 is also upregulated during pathological hypertrophy induced by pressure overload. In cardiomyocytes, genetic overexpression of miR-222 enhances survival, decreases fibrosis, and lessens dysfunction following TAC, whereas suppression of miR-222 induces hypertrophy and heart failure. Mechanistic research revealed that miR-222 directly targets p53-up-regulated modulator of apoptosis (PUMA), Hmbox1, and NFATc3, highlighting its capacity to inhibit apoptosis and maladaptive transcriptional remodeling [158]. Therefore, abnormal hypertrophy is suppressed by miR-222 and modulating it may be a feasible therapeutic approach. In heart illness, miR-128 has recently been identified as a pro-hypertrophic and pro-apoptotic factor. By directly targeting MDFI (MyoD Family Inhibitor) and activating the Wnt/β-catenin pathway, overexpression of miR-128 in cardiomyocytes increases death and decreases proliferation [159]. This implies that, especially in the context of heart failure, miR-128 may drive remodeling by both encouraging cell death and reducing regeneration ability. Moreover, fibrotic remodeling is facilitated by miRNA-221, which is strongly linked to miR-222. Reduced levels of the miR-221/222 family are associated with increased fibrosis, fibroblast activation, and TGF-β/SMAD signaling under pressure overload, indicating a protective, antifibrotic role in structural remodeling [160]. Collectively, these results demonstrate that a network of miRNAs, rather than a single miRNA axis, governs hypertrophic responses. Pro-growth miRNAs such as miR-26a-5p and miR-128 promote hypertrophy and apoptosis through mechanisms involving ADAM17, autophagy, NLRP3 inflammasome, and Wnt/β-catenin signaling. In contrast, anti-growth miRNAs like miR-222 and miR-221 counteract pathological remodeling by inhibiting fibrosis, apoptosis, and maladaptive transcriptional pathways. The balance among these miRNAs determines the extent of hypertrophic expansion and whether remodeling remains adaptive or progresses to a maladaptive state [156]. Similarly, it has been demonstrated that miR-128 targets MDFI and activates Wnt/β-catenin signaling to promote hypertrophy and apoptosis [159]. From a therapeutic standpoint, adjusting this network, either by boosting miR-222/miR-221 or by blocking miR-26a-5p or miR-128, may offer a unique way to regulate cardiac remodeling and stop the development of heart failure [161].

### 3.4. microRNAs Associated with Fibrosis and Apoptosis (miR-29, miR-30, miR-34a)

Cardiac fibrosis and apoptosis are central features of maladaptive remodeling that contribute to the progression of heart failure. Several microRNAs have been identified as key regulators of these processes, including miR-29, miR-30, and miR-34a, which coordinate signaling pathways that influence extracellular matrix deposition, cell survival, and programmed cell death [124]. MiR-29 is one of the most well-described antifibrotic miRNAs. It suppresses excessive ECM deposition by directly targeting genes encoding collagen I and III, fibrillin-1, and elastin [162]. Downregulation of miR-29 has been consistently observed in pressure-overload and post-infarction models and correlates with increased fibrosis and worsening cardiac function [163]. In vivo experiments confirmed that loss of miR-29 expression results in myocardial fibrosis, diastolic dysfunction, and heart failure with preserved ejection fraction. MiR-29 effectively limits ECM accumulation and represents a promising antifibrotic therapeutic target [164]. Members of the miR-30 family regulate both fibrosis and apoptosis. MiR-30 reduces apoptosis in cardiomyocytes under stress by maintaining mitochondrial integrity and repressing pro-apoptotic genes such as BCL2-interacting mediator of cell death (BIM) [165]. Furthermore, miR-30 mitigates fibrotic remodeling by downregulating major pro-fibrotic mediators, including CTGF and TGF-β1 [166]. Loss of miR-30 expression has been associated with maladaptive remodeling in pressure-overload and ischemia–reperfusion conditions, supporting its dual role in apoptosis and fibrosis regulation [167]. In contrast, miR-34a functions as a potent pro-apoptotic miRNA in the heart. It targets PNUTS (PPP1R10), BCL2, and SIRT1 to accelerate cardiomyocyte death, with expression increasing under ageing, oxidative stress, and myocardial infarction [168]. Therapeutically, miR-34a inhibition reduces apoptosis, limits fibrosis, and improves cardiac performance in animal models [169]. Additionally, blocking miR-34a attenuates extracellular matrix deposition and further improves cardiac function, supporting its candidacy as a promising therapeutic target [170]. MiR-29 additionally exerts a significant antifibrotic effect by suppressing collagen and ECM-related genes, and loss of miR-29 promotes myocardial fibrosis, diastolic dysfunction, and heart failure with preserved ejection fraction [164,171]. Both miR-29 and miR-30 have been linked to the modulation of fibrosis, while miR-30 additionally protects against apoptosis, and miR-34a predominantly drives apoptosis and fibrosis [172]. Therefore, therapeutic modulation follows a logical direction: increasing miR-29 may reduce fibrosis, increasing miR-30 may enhance cardiomyocyte survival, while inhibiting miR-34a may prevent excessive remodeling and cell loss. Rather than targeting a single miRNA, multi-miRNA modulation could provide a more comprehensive strategy for controlling adverse remodeling in heart failure.

### 3.5. microRNAs and Metabolic Dysregulation of the Failing Heart

Metabolic remodeling is now recognized as a core feature of the failing myocardium, wherein shifts in substrate utilization, impaired mitochondrial function, and energy-producing pathways contribute to inadequate ATP generation and progressive myocardial dysfunction. Recent evidence indicates that microRNAs (miRNAs) are crucial regulators of these metabolic changes, acting at multiple levels, from glucose uptake and glycolysis, through fatty-acid oxidation and mitochondrial biogenesis, to global energy-homeostasis signaling [84,173]. The potential for miRNAs to serve as biomarkers for metabolic derangement in heart failure (HF) has been underscored by clinical data. Altered circulating or myocardial miRNA profiles have been observed in HF patients, correlating with metabolic and structural derangements even before overt functional decline [92,174]. Experimental studies further substantiate the mechanistic role of miRNAs in metabolic dysregulation. A landmark in vitro investigation showed that under metabolic stress (high glucose and palmitate), suppression of miR-208a in human cardiomyocytes preserved mitochondrial DNA content, maintained expression of mitochondrial and respiratory-chain proteins, and rescued oxidative phosphorylation. In contrast, miR-208a overexpression impaired mitochondrial biogenesis and respiration, indicating that miR-208a acts as a negative regulator of mitochondrial oxidative metabolism under metabolic stress [175]. Beyond mitochondrial biogenesis, miRNAs also regulate mitochondrial quality control and substrate utilization. For example, in chronic heart failure models, miR-21-3p is upregulated and suppresses CPT1A, thereby impairing mitochondrial import of long-chain fatty acids and disrupting mitophagy. This maladaptive alteration exacerbates energy deficiency and oxidative stress [176]. More generally, miRNAs affect the elimination of malfunctioning mitochondria and the preservation of mitochondrial health in cardiomyocytes under stress by targeting important regulators of mitochondrial quality control such as PINK1, Parkin, or FUNDC1 [177]. It has also been shown that miRNAs regulate glucose metabolism. MiR-199a, miR-378, miR-21, miR-133, miR-503, and members of the let-7 family are among the miRNAs that are consistently dysregulated in dilated cardiomyopathy and heart failure, according to a recent overview of research on cardiac glucose metabolism. These miRNAs may be indicators of poor myocardial glucose consumption [178]. Members of the miR-30 family have been shown to enhance fatty-acid β-oxidation under metabolic stress, supporting mitochondrial energy metabolism in preclinical diabetic-heart models. MiR-30 promotes fatty acid beta-oxidation and endothelial cell dysfunction and is a circulating biomarker of coronary microvascular dysfunction in pre-clinical models of diabetes [179]. Therefore, there is growing evidence that deregulation of microRNAs may influence a maladaptive metabolic pattern seen in heart failure, at least partially. For instance, suppression of miR-208a maintained mitochondrial DNA content, mitochondrial markers, and oxidative phosphorylation in human cardiomyocytes under metabolic stress (high glucose and palmitate). In contrast, overexpression of miR-208a reduced mitochondrial biogenesis and respiration, suggesting that miR-208a can function as a negative regulator of mitochondrial oxidative metabolism under metabolic challenge [175]. Furthermore, upregulation of miR-21-3p has been demonstrated to downregulate CPT1A, impede the import of long-chain fatty acids into mitochondria, and worsen mitophagy dysfunction in chronic heart failure models. These alterations compromise the integrity of mitochondria and the ability to oxidize fatty acids, which may contribute to the energy deficit in failing myocardium [176]. These findings support the concept that miRNA dysregulation may impair mitochondrial biogenesis and substrate oxidation in cardiomyocytes. Nevertheless, the proposition that concomitant overexpression of deleterious miRNAs and downregulation of cardioprotective miRNAs (e.g., miR-378 or members of the miR-30 family) drives comprehensive metabolic remodeling in human heart failure has yet to be definitively validated. Evidence from longitudinal in vivo investigations remains scarce, and most mechanistic insights are currently derived from in vitro experiments or preclinical animal models [180]. Likewise, the concept that miRNA-mediated metabolic rewiring precedes overt structural remodeling in the failing myocardium remains speculative, as longitudinal in vivo validation is largely lacking [181]. Consequently, while therapeutic modulation of miRNAs, including strategies such as miRNA mimics, antimiRs, sponges, or exosome-mediated delivery, represents a promising approach to restore metabolic homeostasis, translation to clinical heart failure therapy necessitates rigorous validation, precise tissue-specific delivery, and comprehensive safety evaluation [182]. Taken together, these considerations underscore the necessity for well-designed, longitudinal human studies to delineate the temporal relationship between miRNA dysregulation, metabolic remodeling, and structural progression in heart failure. Only through such studies can the prognostic utility of miRNAs be firmly established, and safe, targeted miRNA-based interventions be developed to correct maladaptive metabolic phenotypes [106].

#### 3.5.1. Discussion of Upregulated vs. Downregulated miRNAs in HF

Metabolic remodeling is a hallmark of the failing heart, characterized by impaired mitochondrial bioenergetics, reduced fatty-acid oxidation, and a shift toward inefficient substrate utilization [183]. Recent evidence implicates microRNAs as major regulators of these maladaptive metabolic processes in heart failure. MiR-30 promotes fatty acid beta-oxidation and endothelial cell dysfunction and is a circulating biomarker of coronary microvascular dysfunction in pre-clinical models of diabetes [179]. Notably, it has been demonstrated that miR-208a is increased in metabolically challenged cardiomyocytes and suppresses mitochondrial biogenesis by targeting the mitochondrial biogenesis complex components, which lowers mtDNA content and oxidative capacity in high-lipid or diabetic situations [175]. In parallel, miR-34a, which is also elevated in pathological states, downregulates SIRT1 and other mitochondrial regulators, promoting mitochondrial dysfunction and energetic decline in cardiac cells [170,184]. Conversely, certain miRNAs are decreased in heart failure yet normally act to support metabolic homeostasis: reduced levels of members of the miR-30 family, which have been associated with enhanced fatty-acid β-oxidation and mitochondrial integrity, contribute to the energy deficit in failing hearts. The result of these changes is a maladaptive miRNA-mediated metabolic network, where a decrease in mitochondrial function, ATP production, and metabolic flexibility is caused by the overexpression of harmful miRNAs (like miR-208a and miR-34a) and the loss of protective ones (like miR-30) [175]. From a therapeutic standpoint, focusing on this network, for example, by blocking miR-208a or miR-34a while boosting miR-30 activity, offers a viable method to enhance bioenergetics, restore metabolic competence, and stop or reverse the progression of heart failure [185]. In addition, emerging data suggests that maladaptive miRNA regulation also impacts mitophagy and mitochondrial quality control. For example, miR-21-3p is upregulated in chronic heart failure and suppresses CPT1A, impairing fatty acid import into mitochondria and exacerbating mitophagy dysfunction in cardiomyocytes [176]. Furthermore, a characteristic of failing myocardium is decreased fatty acid oxidation (FAO), which is exacerbated by unbalanced miRNA expression and leads to lipid buildup, oxidative stress, and further mitochondrial damage. Therefore, adjusting the miRNA balance may enhance mitochondrial turnover and resilience in addition to restoring energy production [185]. 

#### 3.5.2. Functional Consequences of These Changes on Cardiac Contractility, Extracellular Matrix, and Mitochondrial Metabolism

The dysregulation of cardiac miRNAs in heart failure has profound functional consequences on contractile performance, extracellular matrix (ECM) remodeling, and mitochondrial metabolism. Overexpression of miR-208a, for example, has been shown to repress mitochondrial biogenesis and reduce respiratory capacity in human cardiomyocytes under metabolic stress, leading to diminished ATP production and impaired contractile protein expression, which collectively compromise contractile performance [175]. In parallel, miR-34a contributes to maladaptive remodeling by enhancing profibrotic signaling, activating TGF-β/Smad pathways, and promoting ECM deposition through direct and indirect targets, exacerbating interstitial fibrosis and increasing myocardial stiffness [186]. Elevated miR-208a in exosomes from hypoxic cardiomyocytes further stimulates fibroblast activation, collagen I/III expression, and fibroblast proliferation, amplifying fibrotic remodeling and reducing compliance [187]. Dysregulated miRNAs affect both organelle quality control and bioenergetics at the mitochondrial level. Increased activity of the miR-34 family sensitizes cells to apoptotic pathways, causing mitochondrial fragmentation and cell death, whereas loss of mitochondrial biogenesis and decreased expression of mitochondrial ribosomal and integrity-related proteins disturb mitochondrial turnover [188]. These combined effects weaken the heart’s resilience, as the diminished energetic output and ATP generation reduce the capacity to sustain contractile demand, while fibrosis mechanically loads the myocardium and further degrades function [189]. Functionally, this miRNA-driven maladaptive network produces a vicious cycle: fibrosis raises wall stiffness, mitochondrial dysfunction decreases contractile reserve, and both hinder the heart’s capacity to react to increased strain. Restoring mitochondrial function, reversing fibrosis, and eventually revitalizing contractile performance could be achieved through therapeutic approaches targeted at normalizing miRNA expression, such as blocking miR-208a or miR-34a or modifying their transport via exosomes [190].

## 4. Molecular Pathways and Target Genes Regulated by microRNAs

### 4.1. Key Signaling Pathways: PI3K/Akt, TGF-β, NF-κB, Wnt, MAPK

By carefully controlling key signaling pathways that govern hypertrophy, fibrosis, apoptosis, and metabolic function, namely Wnt/β-catenin, TGF-β, NF-κB, MAPK, and PI3K/Akt, microRNAs coordinate cardiac remodeling [191]. Specific miRNAs target multiple nodes within these pathways, functioning as fine-tuners of cardiac homeostasis. For instance, miR-133a and miR-1 suppress components of PI3K/Akt and MAPK signaling to inhibit pathological hypertrophy, stabilize cytoskeletal architecture, and preserve excitation–contraction coupling [192]. miR-21 and miR-29 regulate TGF-β pathway elements, thereby controlling fibroblast proliferation, collagen synthesis, and progression of fibrosis. For example, miR-21 promotes cardiac fibroblast-to-myofibroblast transformation by repressing Jagged1, which amplifies TGF-β1/Smad signaling and enhances collagen deposition [193]. In diabetic cardiomyopathy, upregulation of miR-21 has been linked to fibrosis via TGF-β/Smad, implicating it as a mediator of pathological ECM remodeling [194]. Meanwhile, miR-29 is downregulated in fibrotic conditions. In models of cardiac fibrosis, reduced miR-29 correlates with increased expression of collagen and other ECM proteins, while experimental restoration of miR-29 suppresses Smad3-dependent TGF-β signaling and attenuates fibrosis [195]. NF-κB signaling is modulated by miR-146a-5p, which dampens inflammatory and pro-fibrotic responses; in a model of isoproterenol-induced cardiac fibrosis, its overexpression suppressed FGF2, reducing collagen I and α-SMA levels, and it restrains NF-κB via negative feedback to limit inflammatory gene expression and cellular stress [196]. Additional research suggests that miR-146a helps to restrain the NF-κB pathway via negative feedback, thus reducing inflammatory gene expression and limiting cellular stress [197]. Additionally, members of the miR-30 family alter signaling crosstalk via controlling Wnt/β-catenin activity, which impacts calcium homeostasis, mitochondrial metabolism, and cardiomyocyte proliferation. Since these miRNAs typically support metabolic balance and structural resilience, dysregulation of miR-30 is linked to poor mitochondrial function and maladaptive cardiac remodeling [198]. MiRNAs mediate crosstalk between signaling pathways to amplify or buffer cellular responses; for example, miR-34a can promote maladaptive remodeling and TGF-β-mediated fibrosis by suppressing PI3K/Akt survival signaling and upregulating Smad4. Similarly, miR-208a, secreted in exosomes from stressed cardiomyocytes, affects hypertrophic development through MAPK and Akt pathways and stimulates fibroblast activation, myofibroblast differentiation, and collagen I/III expression [199]. Similarly, miR-208a is secreted in exosomes from stressed cardiomyocytes and affects hypertrophic development through the MAPK and Akt pathways. By transferring miR-208a to cardiac fibroblasts, these exosomes can efficiently link paracrine-driven fibrosis to cardiomyocyte stress by stimulating fibroblast activation, myofibroblast differentiation, and collagen I/III expression [187]. Through this network, miRNAs act as master regulators, integrating signals of growth, survival, metabolism, inflammation, and intercellular communication; when their balance is disrupted, homeostasis is lost, leading to maladaptive remodeling, contractile dysfunction, fibrosis, and progression to heart failure. For example, miR-146a-5p is downregulated in fibroblasts under fibrotic stress; restoration of miR-146a-5p directly targets FGF2, reduces collagen I and α-SMA expression, and attenuates fibroblast activation. Therapeutic modulation of these miRNAs, such as inhibiting miR-34a or miR-208a, or restoring miR-146a-5p, may help rebalance signaling, reduce fibrosis, and preserve cardiac function [196].

### 4.2. Interactions Between miRNAs and Transcription Factors (e.g., NFAT, GATA4)

Transcription factors (TFs) and microRNAs (miRNAs) form closely interconnected regulatory circuits that govern cardiac stress adaptation, hypertrophic signaling, and pathological remodeling. TFs directly regulate miRNA transcription, while miRNAs fine-tune TF expression, generating feedback and feedforward loops that stabilize cardiac gene programs [200]. For example, miR-1 suppresses hypertrophic growth by targeting GATA4 and MEF2A and by dampening calcium calcineurin NFAT signaling [201]. Conversely, NFATc3 induces miR-23a transcription, creating a prohypertrophic loop [202]. Systems-level analyses further highlight the broad presence of coordinated miRNA TF networks in heart disease [203].

NFAT signaling represents a central hub in pathological hypertrophy. miR-31-5p attenuates NFAT-driven gene expression by targeting Nfatc2ip and reducing β-MHC levels [200]. Similarly, miR-424/322 limits hypertrophy and fibrosis by targeting NFATc3 and furin in angiotensin II-induced stress, while NFATc3 can transcriptionally activate miR-424/322, establishing a regulatory feedback loop [204]. In contrast, the lncRNA Jpx enhances hypertrophic responses by sponging miR-145-5p, thereby relieving repression of NFATc3 and strengthening NFAT signaling [205]. Dysregulation of these interconnected miRNA NFAT circuits contributes to maladaptive remodeling and heart failure progression [206].

GATA4 is another essential TF that regulates cardiomyocyte survival and structural gene expression under stress [207]. Its activity is tightly controlled by miRNAs and circular RNAs. CircRNA_000203 promotes hypertrophy by sponging miR-26b-5p and miR-140-3p, thereby preventing repression of Gata4 [208], whereas direct overexpression of miR-26b reduces GATA4 levels and attenuates hypertrophic growth [207]. In fibrotic remodeling, circ-sh3rf3 modulates a GATA4 related pathway that increases miR-29a expression and limits fibroblast activation [209]. Together, these multilayered interactions between miRNAs, TFs, and noncoding RNAs illustrate a finely tuned regulatory network that determines whether cardiac remodeling remains adaptive or becomes maladaptive.

### 4.3. Crosstalk Between miRNAs and Other Non-Coding RNAs (lncRNAs, circRNAs)

Non-coding RNAs (ncRNAs), which include circular RNAs (circRNAs), long non-coding RNAs (lncRNAs), and microRNAs (miRNAs), form a multilayered regulatory network that plays a critical role in controlling gene expression in the heart. An increasing amount of research suggests that lncRNAs and circRNAs can function as competing endogenous RNAs (ceRNAs), or “sponges,” for miRNAs, modifying the availability of miRNAs to their mRNA targets and impacting cardiac remodeling processes such as fibrosis, hypertrophy, and heart failure [210]. Beyond circRNA_000203, other circRNAs likewise engage in ceRNA networks influencing cardiac pathophysiology. For instance, in ischemic heart failure patients, global circRNA–miRNA–mRNA interaction mapping identified hundreds of potential ceRNA axes, some experimentally validated; a network centered on circBPTF was shown to sponge multiple miRNAs, thereby derepressing mRNA targets involved in cardiovascular disease-related pathways [211]. Thus, circRNAs provide an additional epigenetic layer of regulation, complementing miRNA-mediated control, by dynamically shaping miRNA availability and thereby modulating transcription factor expression, signaling cascades, and structural gene regulation in cardiac cells [212]. Given their stability, tissue-specific expression, and functional potency, these circRNA–miRNA–mRNA/lncRNA–miRNA–mRNA axes represent promising candidates both for biomarkers and as therapeutic targets in pathological cardiac remodeling, hypertrophy, fibrosis, and heart failure [213]. Recent studies have highlighted that ncRNAs form complex ceRNA networks that exert both pathogenic and protective roles in cardiac remodeling. For instance, circHIPK3 has been shown to promote cardiac fibrosis by sponging miR-29b-3p, thereby derepressing its targets such as COL1A1 and COL3A1, leading to enhanced fibroblast proliferation and extracellular matrix deposition [214]. Similarly, the long non-coding RNA MALAT1 modulates fibroblast activation post-myocardial infarction by sequestering miR-145, preventing inhibition of the TGF-β1/Smad signaling pathway and exacerbating fibrotic remodeling [215]. Conversely, certain circRNAs, such as circPan3, act protectively by sponging miR-320-3p, derepressing HSP20, and attenuating hypertrophic responses under adrenergic stress conditions [216].

### 4.4. Network-Based Understanding of miRNA Regulation in Cardiac Remodeling

Cardiac remodeling is not merely the consequence of isolated miRNA–mRNA interactions, but rather the outcome of complex, multilayered regulatory networks in which microRNAs (miRNAs), transcription factors (TFs), and non-coding RNAs (ncRNAs), such as long non-coding RNAs (lncRNAs) and circular RNAs (circRNAs), coordinate to regulate gene programs implicated in hypertrophy, fibrosis, metabolic adaptation, and cell fate decisions [210]. System-level analyses demonstrate that miRNAs and TFs often participate in feed-forward or feedback loops, wherein a TF regulates miRNA expression, and the miRNA in turn modulates the same or complementary targets. This architecture enables fine-tuned, robust, and context-dependent regulation of gene expression in cardiac disease. Network reconstruction in patients with heart failure identified 26 miRNAs and 22 TFs forming interconnected regulatory loops controlling key cardiac genes [203]. Additionally, competing endogenous RNA (ceRNA) networks involving lncRNAs and circRNAs add a further regulatory layer. circRNAs and lncRNAs act as molecular sponges for miRNAs, reducing their availability and thereby derepressing mRNA or TF targets. Such ceRNA interactions have been implicated in pathological cardiac remodeling, including hypertrophy and fibrosis [212]. Recent omics-based studies reveal that miRNA–mRNA networks also modulate cardiac metabolic remodeling. Specific miRNAs target enzymes and proteins critical for β-oxidation and oxidative phosphorylation, coordinating the metabolic shift from fetal to postnatal life and supporting energetic homeostasis in stressed myocardium [217]. Network reconstructions allow identification of hub miRNAs, which are central nodes critical for maintaining cardiac homeostasis. Dysregulation of these hub miRNAs is associated with maladaptive remodeling, including fibrosis, hypertrophy, and inflammation [211]. Mapping ceRNA modules specific to pathological phenotypes enables targeted interventions at the network level rather than single-gene targeting, potentially minimizing off-target effects and improving therapeutic efficacy [213].

## 5. microRNAs as Biomarkers of Heart Failure

In recent years, investigators have tested blood-based biomarkers for the purpose of identifying heart failure. miRNAs have emerged as promising biomarkers due to their stability in biofluids, tissue specificity, and regulatory involvement in cardiometabolic, inflammatory, and fibrotic pathways [218]. A variety of circulating analytes, including natriuretic peptides, cardiac injury markers, inflammatory and fibrotic biomarkers, metabolic and renal indicators, as well as emerging molecular signatures such as circulating microRNAs and extracellular vesicles, have been interrogated towards this end [219]. Unlike traditional protein biomarkers, miRNAs reflect upstream molecular alterations, offering insight into the early and dynamic processes driving HF onset and progression. 

### 5.1. Circulating microRNAs in Plasma and Serum

**Figure 3:**
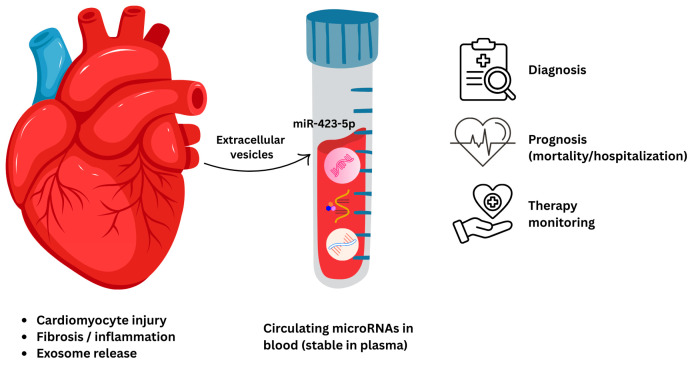
Circulating microRNAs as biomarkers of heart failure. Cardiac injury, fibrosis, and inflammation promote the release of microRNAs from cardiomyocytes via extracellular vesicles into the bloodstream, where they remain stable. Circulating microRNAs can be used for diagnosis, prognostic stratification, and therapy monitoring in heart failure.

Circulating miRNAs originate from a variety of cellular processes, and their presence in plasma and serum reflects both physiological signaling and pathological injury within the myocardium and vasculature [220,221,222,223]. miRNAs are transcribed as primary transcripts, processed in the nucleus via Drosha, and further cleaved by Dicer to form mature miRNAs, which are then incorporated into the RNA-induced silencing complex (RISC). While miRNAs predominantly function intracellularly, a significant fraction is exported into the bloodstream, where they exert paracrine or endocrine-like effects [222,223]. miRNAs are released into circulation through several mechanisms, each reflecting different states of myocardial health and injury (Figure 3). Active secretion pathways play a fundamental role in the regulated release of miRNAs [224]. Cardiomyocytes, fibroblasts, immune cells, and endothelial cells can pack miRNAs into exosomes, microvesicles or apoptotic bodies, which serve as stable carriers that protect miRNAs from enzymatic degradation [104].

Exosomal miRNAs, specifically, have gained interest due to their cargo composition that is selectively regulated and reflects the molecular state of their parent cells. This selective packing is mediated by RNA-bonding proteins such as hnRNPA2B1, AGO2, and ELAVL1, suggesting that circulating miRNAs represent a curated snapshot of cellular gene expression [225,226,227,228]. In addition to vesicular transport, miRNAs can circulate bound to high-density lipoproteins (HDL), Argonaute2 (Ago2), and nucleophosmin 1 (NPM1) complexes [229,230]. These connections may enable tailored distribution to recipient cells and offer additional resistance to RNAse action. Changes in HDL-miRNA profiles have been reported in heart failure and cardiometabolic disease, and HDL-associated miRNAs seem to be involved in lipid homeostasis and vascular control [231,232].

The second substantial contributor to circulating miRNA levels during myocardial injury is passive release mechanisms. In conditions involving ischemia, oxidative stress, mechanical stress, or inflammation, necrotic or damaged cardiomyocytes release intracellular miRNAs directly into the extracellular space [233,234]. Classic examples are the release of miR-1, miR-133a, miR-499, and miR-208 during acute injury of the myocardium. These miRNAs function as biomarkers of cardiomyocyte membrane disruption, with kinetics that may supplement or even precede traditional protein injury markers such as troponins [57,235,236,237]. Beyond the heart, circulating miRNAs also result from renal failure, vascular remodelling, systemic inflammation, and metabolic stress, which are prevalent in heart failure [58,238,239,240]. The complicated yet instructive circulating miRNA landscape that results from the interaction of different sources reflects the multifaceted pathophysiology of heart failure. Unlike natriuretic peptides, which primarily reflect hemodynamic stress, miRNAs can provide molecular signals related to fibrotic remodeling, inflammation, myocardial injury, and neurohormonal activation [241,242]. Patients who arrive with new or worsening heart failure regularly have higher levels of miRNAs [243]. miR-423-5p, one of the earliest identified HF-associated miRNAs, has been proposed as a diagnostic marker capable of distinguishing HF-related dyspnea from non-cardiac causes [241]. Similar to this, high-sensitivity troponin tests may be enhanced by miR-1, miR-133a, miR-208a/b, and miR-499, which increase dramatically after cardiomyocyte injury, especially in individuals with overlapping HF and ischemic presentations [244]. MiRNAs linked to fibrosis are also relevant for diagnosis. Heart failure is associated with an increase in miR-21, which is highly expressed in cardiac fibroblasts and is linked to unfavorable remodelling and extracellular matrix expansion [116]. Members of the miR-29 family (miR-29a/b/c), which control collagen production, show changed levels in HF and may provide diagnostic information about fibrotic load [245]. Early in the development of HF, several miRNAs linked to energy metabolism and mitochondrial dysfunction are changed [246]. For instance, cardiometabolic HF phenotypes frequently exhibit dysregulation of miR-133a, a regulator of GLUT4 and myocardial glucose uptake [247]. When oxygen transport is compromised, hypoxia-related miRNAs, such as miR-210, rise and can be used as markers of myocardial stress during HF flare-ups [248,249]. The diagnostic performance of individual miRNAs is promising, but miRNA panels generally outperform single markers [250]. Combinatorial signatures that integrate multiple miRNAs—representing structural injury, metabolic stress, and inflammation—have been shown to enhance diagnostic accuracy and may eventually be paired with established biomarkers to improve the screening strategies [251]. The identification of low-abundance miRNAs has been made possible by developments in digital PCR and high-throughput sequencing, expanding the pool of potential candidates for clinically significant diagnostic functions [252]. All things considered, circulating miRNAs constitute a new class of diagnostic instruments that can enhance or supplement existing HF biomarker approaches. They may serve as early warning indications for both acute decompensation and the progression of chronic heart failure due to their capacity to detect upstream molecular alterations.

#### miRNA Profiles Across HF Phenotypes

Distinct miRNA signatures have been documented across heart failure phenotypes, particularly in comparing HF with preserved ejection fraction (HFpEF) and HF with reduced ejection fraction (HFrEF). Circulating miRNAs can act as molecular fingerprints to differentiate between these syndromes because they are caused by distinct pathophysiological mechanisms: HFrEF is more closely associated with cardiomyocyte loss and structural remodelling, while HFpEF is primarily driven by inflammation, endothelial dysfunction, and microvascular disease [253]. Circulating miRNA patterns in HFpEF patients are enriched in pathways associated with metabolic impairment, cardiac enlargement, vascular stiffness, and systemic inflammation. Increased levels of miR-146a, a regulator of the NF-κB pathway, indicate endothelial activation and persistent low-grade inflammation, two characteristics of HFpEF [86]. In HFpEF cohorts, miR-181a, miR-30a-5p, and miR-199b-5p have also been found, suggesting abnormal energy metabolism, poor calcium management, and fibrosis. Notably, HFpEF appears to have higher levels of miRNAs linked to macrophage polarisation, such as miR-34a and miR-223, which is consistent with the immune–metabolic profile of this phenotype [58,105]. On the other hand, miRNAs indicating cardiomyocyte damage, myocyte apoptosis, reduced contractility, and structural remodelling are indicative of HFrEF. These include miR-1, miR-133a, miR-208a/b, and miR-499, which are released when myocardial injury occurs either acutely or over time. Furthermore, persistent fibrosis and remodelling that contribute to increasing systolic dysfunction are indicated by often raised miR-21 and miR-29 [116]. Certain miRNAs can successfully distinguish between HFpEF and HFrEF, according to comparative assessments. For instance, while injury-related miRNAs like miR-1 and miR-499 are more characteristic of HFrEF, larger levels of miR-146a and miR-181a favour an HFpEF phenotype. The use of miRNAs as molecular classifiers may benefit from these variations, especially as HF phenotyping becomes more important for tailored treatments [125].

### 5.2. Correlation Between miRNA Levels and Disease Severity (NYHA Class, Ejection Fraction)

Circulating microRNAs show significant potential as biomarkers of heart failure severity, with numerous studies demonstrating clear associations between specific miRNA levels and established clinical measures such as NYHA functional class and left ventricular ejection fraction (LVEF) [251,254]. Circulating miRNA studies remain methodologically heterogeneous, with variability in cohort composition, biospecimen handling, analytical platforms, and statistical adjustment. The strength of evidence supporting circulating miRNAs in heart failure varies considerably. miR-423-5p is among the most consistently validated candidates, with replication across multiple independent cohorts, supportive meta-analytic data, and some studies suggesting incremental value beyond natriuretic peptides. miR-21 and miR-126 are also supported by replication and strong biological plausibility, although their incremental prognostic utility over established biomarkers remains inconsistent. miR-122 shows growing multi-cohort support but is likely influenced by systemic metabolic processes rather than cardiac-specific pathology. In contrast, miR-146a and miR-210 currently rely on more limited cohort validation without meta-analytic confirmation or clear demonstration of additive clinical value. Collectively, these distinctions highlight the importance of differentiating widely studied miRNAs from those with reproducible and clinically actionable evidence [58,86,92,105,248,252,255,256]. Table 2 summarizes design features that directly influence reproducibility and clinical interpretability.

Notably, only a subset of candidate miRNAs demonstrate reproducible associations across adequately adjusted cohorts, underscoring the need for standardized analytical workflows and external validation before routine clinical adoption can be considered.

The concentrations of miRNAs in plasma and serum frequently fluctuate in tandem with the physiological and structural decline typical of progressive heart failure because they reflect upstream molecular processes, such as cardiomyocyte injury, fibrosis, inflammation, endothelial dysfunction, and energetic remodelling [59,116]. As symptoms develop, the concentration of many miRNAs rises. Among these, miR-423-5p has shown a stepwise rise from mild to advanced NYHA classes. It correlates with both haemodynamic congestion and symptom burden, making it one of the most consistently raised markers [86,255]. Similarly, miR-21, a key regulator of fibroblast activation and extracellular matrix deposition, increases in parallel with deteriorating functional capacity and more extensive cardiac remodeling [117,243]. As the NYHA class worsens, injury-associated miRNAs such as miR-208, miR-499, and miR-133a also increase, indicating increased cardiomyocyte stress and membrane disruption. Patients with advanced symptoms exhibit greater circulating levels of hypoxia-responsive miRNAs, especially miR-210, which is consistent with reduced oxygen utilisation and microvascular dysfunction in severe heart failure [92,248].

On the other hand, as heart failure worsens, other miRNAs decline. Reduced circulating concentrations of miR-150 are linked to greater neurohormonal activation, increased inflammation, and poor functional status, making them one of the most consistent inverse relationships with NYHA class [258]. As the severity of the disease increases, endothelium-enriched miRNAs like miR-126 also decrease, reflecting worsening endothelial dysfunction and decreased exercise tolerance. Members of the miR-30 family, which contribute to mitochondrial stability and protection against apoptosis, demonstrate reduced levels in advanced disease, indicating increased energetic stress in severe HF [256,259,260].

Additionally, circulating miRNAs and LVEF are strongly correlated. Several miRNAs exhibit an inverse association with systolic function, meaning that progressively lower ejection fraction is correlated with greater circulating levels [252]. In both ischaemic and non-ischaemic cardiomyopathy, increased miR-21 is associated with fibrotic remodelling and ventricular stiffness, both of which lead to systolic dysfunction [115,118]. Patients with falling LVEF also have higher plasma concentrations of the miR-29 family, which regulates collagen synthesis and is consistent with continuous extracellular matrix turnover. Similar inverse correlations with LVEF are shown by other miRNAs linked to hypertrophic remodelling and reduced contractility, such as miR-125b, miR-199a, and miR-208b [261,262]. On the other hand, improved systolic function is favourably correlated with several miRNAs. Patients with severe systolic dysfunction typically have reduced circulating levels of miR-1 and miR-133a, which are both implicated in calcium management and electrical stability [58,263]. This is likely due to their intracellular depletion during structural and electrophysiological instability. Poor ventricular performance is also correlated with lower systemic levels of miR-30 and miR-126, which indicate endothelial and mitochondrial degradation [252,257].

There is some phenotypic dependence in these connections. Since systolic failure in HFrEF is primarily caused by cardiomyocyte loss and structural remodelling, miRNAs linked to myocyte injury, such as miR-1, miR-133a, miR-208, and miR-499, show the highest correlations with LVEF reduction [58,119]. Instead of the ejection fraction itself, which is maintained in HFpEF, markers including miR-21, miR-146a, and miR-181a are more strongly correlated with diastolic dysfunction, microvascular inflammation, and increased myocardial stiffness [123,141].

Studies analysing combinations of miRNAs demonstrate that multimarker signatures correlate more strongly with illness severity than single miRNAs alone [125]. Incorporating markers of myocardial damage, fibrotic remodelling, inflammation, and metabolic stress into integrated profiles can improve early identification of patients at risk for rapid development and more accurately parallel both NYHA class and LVEF [126]. These findings have significant clinical ramifications. Circulating miRNAs provides an extra quantitative layer to evaluate disease severity because LVEF does not fully represent myocardial performance and because NYHA class might be subjective and impacted by comorbidities; several studies show that miRNA-based signatures add independent and incremental prognostic value beyond conventional clinical and biomarker indices [126,264]. As miRNA quantification becomes more standardized and robust reference ranges are developed, circulating miRNAs hold considerable promise as objective molecular tools to evaluate HF severity and guide personalized management strategies.

### 5.3. Diagnostic and Prognostic Value of miRNAs in Early HF Detection and Therapy Monitoring

Between traditional biomarkers and the underlying biology of heart failure, circulating miRNAs occupy an intriguing space. Many of them monitor processes like hypertrophy, fibrosis, apoptosis, angiogenesis, and metabolic remodelling that occur before changes in natriuretic peptides or even echocardiographic parameters. They are released from cardiomyocytes, fibroblasts, endothelial cells, and immune cells in response to stress [58,86]. They are, therefore, appealing options for heart failure (HF) early diagnosis and dynamic monitoring. In terms of diagnosis, several studies demonstrate that miRNA signatures can identify HF more precisely and earlier than single protein biomarkers. According to reviews and cohort data, HF is associated with changes in myocyte-injury-related miRNAs (miR-1, miR-133a, miR-208, miR-499), fibrosis-related miRNAs (miR-21, miR-29 family), and inflammation/endothelial miRNAs (miR-146a, miR-126, miR-181a) [58]. Crucially, these alterations are not exclusive to advanced illness. Even when natriuretic peptides are only slightly elevated, and LVEF is still maintained, changes in circulating miRNAs have been observed in patients with risk factors, asymptomatic LV dysfunction, or early HFpEF [265]. Particularly in HFpEF, a multi-miRNA panel that included endothelial and inflammation-related miRNAs outperformed individual miRNAs and enhanced the ability to distinguish between HFpEF and non-HF controls [125]. The data is becoming more consistent for prognostic stratification. Abnormal circulating miRNA levels were linked to increased all-cause and cardiovascular mortality in established HF as well as a higher risk of incident HF in at-risk populations, according to a large meta-analysis of individual patient data [126]. Additionally, this study demonstrated that miRNA combinations outperformed single markers, supporting the notion of multi-miRNA “risk signatures” as opposed to depending on a single molecule. Similarly, even after adjusting for natriuretic peptides and clinical covariates, higher levels of injury- and fibrosis-related miRNAs tracked worse NYHA class, lower LVEF, more advanced remodelling, and higher rates of hospitalisation and death in multiple cohorts summarised in a dedicated review on the diagnostic and prognostic relevance of circulating miRNAs in HF [265]. The transition from relative expression to population-based reference values is another step towards clinical application. For a panel of HF-related miRNAs (including miR-1, miR-21, miR-29a, miR-126, and miR-423-5p) in both healthy individuals and HF patients, a recent Biomedicines study established whole-blood reference ranges. The study also demonstrated that deviations from these reference intervals were linked to the presence and severity of HF [252]. If miRNAs are to be interpreted in routine laboratory settings similarly to troponin or NT-proBNP, rather than just as research tools, this kind of work is crucial. MiRNAs show promises for tracking the effectiveness of treatment as well. Changes in miRNA levels during therapy can serve as an early indicator of reverse remodelling because they represent upstream biology. Brioschi et al. demonstrated that several circulating miRNAs involved in hypertrophy, fibrosis, and apoptosis were significantly altered over follow-up in patients with HFrEF treated with sacubitril/valsartan; some of these changes coincided with improvements in haemodynamics and functional capacity, indicating that drug-induced remodelling is at least partially mediated, and measurable, via miRNA pathways [261]. MiRNA patterns that differentiate responders from non-responders and predict reverse remodelling or persistent dysfunction are also described in reviews that summarise data from cardiac resynchronisation therapy, LVAD implantation, and guideline-directed medical therapy [86,107]. In this way, serial miRNA profiling could be used in conjunction with natriuretic peptides and imaging to improve choices about advanced therapy referral, drug uptitration, and device implantation. A few useful themes show up in all of these studies. First, multi-marker strategies consistently outperform single miRNAs. Compared to single miRNAs, panels combining injury, fibrosis, inflammatory, and metabolic miRNAs usually exhibit higher AUCs for HF diagnosis and better calibration with NYHA class and LVEF. Some panels retain prognostic value even after adjusting for age, renal function, natriuretic peptides, and troponin [125,126,251,266]. Second, HFpEF (preserved EF but abnormal diastolic function and microvascular inflammation), early or subclinical HF, and complex multimorbid patients whose NYHA class is significantly influenced by lung disease, obesity, or frailty rather than pure cardiac status are examples of situations where miRNAs appear to be especially helpful [108,123]. Lastly, current diagnostic reviews highlight the likelihood that miRNAs will be incorporated into more comprehensive biomarker strategies rather than taking the place of current tests: panels that include natriuretic peptides, high-sensitivity troponin, and imaging markers of strain or fibrosis may provide the most complete picture of disease activity and progression [127,219]. Notwithstanding the potential, there are still issues with clinical translation. It is challenging to compare cut-offs across studies due to pre-analytical variation (plasma vs. serum, haemolysis, storage conditions), analytical platforms (qPCR vs. sequencing), and normalisation techniques. Sex-specific and HFpEF-specific data are just now beginning to appear, and many cohorts are small, single-center, and enriched for HFrEF [221,265]. However, the path forward is evident: more recent research consistently demonstrates that miRNA signatures can enhance early HF detection, improve prognostic stratification, and provide a dynamic, mechanistically based means of tracking therapy response in both HFrEF and HFpEF, particularly when employed as part of multimodal, precision-medicine approaches.

## 6. Comparison with Conventional Biomarkers (BNP, NT-proBNP, Troponins)

### 6.1. Sustainability and Detectability of miRNAs in Body Fluids

Circulating microRNAs (miRNAs) have attracted considerable attention as cardiovascular biomarkers because of their high molecular stability and consistent detectability in body fluids when compared with conventional protein biomarkers such as BNP, NT-proBNP, and cardiac troponins [267]. Despite their small size, miRNAs are protected from RNase-mediated degradation through their association with extracellular vesicles, RNA-binding proteins such as Argonaute-2, and lipoprotein complexes [250]. Multiple experimental studies have demonstrated that circulating miRNAs remain stable in plasma and serum under conditions that significantly affect protein biomarkers, including prolonged storage and repeated freeze–thaw cycles [268]. In contrast, BNP and NT-proBNP concentrations are influenced by numerous non-cardiac variables such as age, sex, renal dysfunction, obesity, and atrial fibrillation, which may complicate their interpretation in heterogeneous heart failure populations [109,269]. Similarly, high-sensitivity cardiac troponins represent the gold standard for detecting myocardial injury but primarily reflect cardiomyocyte necrosis or chronic low-grade injury rather than specific molecular pathways such as fibrosis, inflammation, or maladaptive remodeling [270]. In contrast, circulating miRNA reflects active gene regulatory processes and therefore provide mechanistic insight beyond that offered by conventional protein biomarkers [103].

### 6.2. Potential for miRNA-Based Diagnostic Panels

Although individual circulating miRNAs have been associated with cardiovascular diseases, increasing evidence indicates that multi-miRNA panels can provide improved diagnostic and prognostic performance compared with single biomarkers alone in heart failure. A recent review and meta-analysis encompassing 45 studies demonstrated that panels of multiple circulating miRNAs could detect heart failure subgroups with acceptable accuracy, with a minimal panel yielding an area under the curve (AUC) ≈ 0.86 for chronic heart failure identification [271]. One of the key advantages of miRNA panels lies in their ability to capture multiple pathophysiological pathways simultaneously, including cardiac hypertrophy, fibrosis, inflammation, endothelial dysfunction, and metabolic remodeling, which cannot be fully characterized by traditional biomarkers such as BNP, NT-proBNP, or troponins alone [267]. For example, specific miRNA signatures have been associated with differences in sympathetic activation, extracellular matrix turnover, and cardiomyocyte stress responses, making them mechanistically informative in addition to diagnostically relevant [86]. Recent work has started to focus on distinct panels tailored for heart failure subtypes. In a study designed to discriminate between HFpEF and HFrEF patients, a panel of circulating miRNAs—including members such as let-7b-5p, let-7e-5p, miR-21-5p, and miR-140-3p—significantly differentiated HFpEF cases from healthy controls and HFrEF patients, illustrating the potential of miRNA panels in benchtop-to-bedside validation for subtype diagnosis. Diagnostic discrimination miRNA panels have also demonstrated prognostic and risk-stratification utility in heart failure. Comprehensive meta-analyses of individual patient data, using standard survival analyses such as Kaplan–Meier estimates, have identified panels of miRNAs predictive of adverse outcomes, including all-cause mortality and cardiovascular death [271]. For example, specific panels consisting of miR-27a-3p, miR-129-5p, miR-145-5p, and miR-590-3p were associated with increased risk of all-cause mortality in HFrEF patients, whereas miR-122-5p and miR-423-5p predicted cardiovascular death, and miR-19a-3p predicted mortality risk in HFpEF patients [238]. Further analyses revealed larger multi-miRNA panels that improve risk stratification and may predict hospitalization or adverse outcomes more accurately than individual miRNAs alone [219]. Different reviews confirm that several circulating miRNAs, including miR-423-5p, miR-122, and miR-21, consistently correlate with heart failure progression and mortality across multiple cohorts [130,254,272]. These findings underscore that miRNA panels can capture complex pathophysiological mechanisms beyond traditional biomarkers, supporting their potential role in comprehensive prognostic assessment in both HFrEF and HFpEF populations [273,274].

## 7. Therapeutic Applications and Future Perspectives of microRNAs in Heart Failure

### 7.1. Therapeutic Concepts and Strategies

A leading translational example of miRNA-directed therapy in heart failure is anti-miR-132 (CDR132L) [110]. The miR-132/212 cluster has been mechanistically linked to adverse cardiac remodeling, cardiomyocyte hypertrophy, and impaired autophagy: initial mechanistic work showed that miR-132/212 are upregulated by hypertrophic stimuli and that overexpression promotes pathological cardiomyocyte growth partly via suppression of the autophagy-promoting transcription factor FOXO3 [111]. To date, only a limited number of miRNA-based therapeutic strategies have progressed beyond preclinical proof-of-concept, with anti-miR-132 representing the most advanced example.

Despite strong mechanistic rationale, therapeutic translation of miRNA modulation remains constrained by delivery efficiency, tissue specificity, and safety considerations. The table below summarizes key features influencing drug-development readiness, including oligonucleotide chemistry, delivery strategies, clinical stage, and known barriers to implementation (Table 3).

Overall, current evidence suggests that successful miRNA therapies will require targeted delivery to the heart, better control of unintended effects on other genes, and careful selection of patients using biomarkers. Without these advances, widespread clinical use is unlikely in the near future.

Following that, preclinical research advanced to a targeted antisense strategy: logically constructed locked-nucleic-acid (LNA) antisense oligonucleotides against miR-132 showed strong cardioprotective benefits in mouse models, reversing hypertrophy caused by pressure overload and enhancing cardiac function [110]. These mechanistic and small-animal data were extended to clinically relevant large-animal models: intravenous dosing of the LNA antimiR (CDR132L) improved systolic and diastolic performance and reduced fibrosis in a pig model of chronic post-MI heart failure [112]. A first-in-human, randomized, double-blind, placebo-controlled Phase 1b study of CDR132L assessed pharmacology, safety, and early human target engagement. The drug was found to be well tolerated with linear, dose-proportional pharmacokinetics and no dose-limiting toxicities in the trial (NCT04045405), which tested ascending single and repeat intravenous doses. Notably, patients receiving ≥1 mg/kg showed sustained, dose-dependent reductions in circulating miR-132 and exploratory declines in NT-proBNP, suggesting biological activity and possible positive effects on remodeling biomarkers [113]. Subsequent translational work consolidated the preclinical-to-clinical bridge: mechanistic, PK/PD, and safety data were combined to support larger efficacy testing and informed an optimized dosing strategy and imaging biomarker selection for clinical trials [275]. Subsequent controlled large-animal studies have confirmed that monthly intravenous administration of CDR132L significantly improves myocardial contractility and reverses adverse remodeling: in a pig model of chronic post-MI heart failure, repeated dosing ameliorated left ventricular (LV) and left atrial (LA) strain parameters, reduced interstitial fibrosis, and correlated with improved ejection fraction and NT-proBNP dynamics [276]. The evidence base has been strengthened by more recent publications and trial reports: a multi-center, randomized Phase II proof-of-concept trial (HF-REVERT, NCT05350969) was initiated to assess repeated intravenous dosing (5 mg/kg and 10 mg/kg) versus placebo in approximately 280 post-MI patients with reduced ejection fraction, using change in LV end-systolic volume index at six months as the primary endpoint [114]. Our molecular understanding of miR-132 suppression in heart failure has been expanded by complementary experimental research reported in 2024–2025. Systemic doses of antimiR-132 significantly reduced profibrotic remodeling in a two-hit animal model of HFpEF: the treatment reduced myocardial collagen buildup, decreased Smad3 phosphorylation downstream of TGF-β1, and improved diastolic dysfunction. Mechanistically, TGF-β1-induced collagen synthesis and Smad3 activation were reduced when miR-132 was inhibited in cardiac fibroblasts, indicating a direct involvement of the miR-132/Smad3 axis in extracellular matrix remodeling [281]. Taken together, the accumulated evidence delineates a coherent bench-to-bedside translational trajectory for miR-132 inhibition in heart failure. This trajectory begins with the initial identification of the miR-132/212 cluster as a maladaptive regulator of cardiomyocyte hypertrophy and autophagy [282], followed by the rational development and preclinical validation of LNA-based antisense inhibitors that demonstrated robust target engagement and functional rescue in rodent models [112]. Subsequent large-animal studies have provided reproducible evidence of efficacy, including improvements in myocardial strain, reductions in interstitial fibrosis, and favorable biomarker modulation, thereby strengthening mechanistic and translational confidence [112]. The successful completion of a first-in-human, randomized, placebo-controlled study further established the safety profile, pharmacokinetic predictability, and sustained target suppression associated with miR-132 antagonism [101,113]. Collectively, these milestones have directly supported the initiation of a Phase II clinical development program, positioning anti-miR-132 therapy as one of the most advanced and potentially disease-modifying, sequence-based therapeutic strategies in contemporary heart failure management [102].

### 7.2. Challenges in Clinical Translation

Despite encouraging preclinical efficacy, the development of miRNA-based therapies for heart failure faces several major translational hurdles. First and foremost is the challenge of delivery: efficient, safe, and tissue-specific delivery systems remain a critical bottleneck. miRNA oligonucleotides are inherently unstable in the circulation (due to nuclease degradation) and achieving sufficient uptake in target cardiac cells without accumulation in off-target tissues requires sophisticated delivery vehicles [283]. Second, off-target effects are a persistent concern. Because individual miRNAs can regulate hundreds of transcripts, partial binding to unintended mRNAs can result in widespread transcriptome perturbations [277]. This pleiotropy complicates dose selection and increases the risk of toxicity, especially in non-cardiac tissues [278]. Third, the clinical application of miRNA therapies may be restricted by immune activation. Synthetic miRNA mimics or inhibitors, particularly when delivered via lipid nanoparticles or viral vectors, may trigger innate immune responses through pattern-recognition receptors (PRRs) such as TLR7/8 or via complement activation, raising a substantial barrier to clinical application [279]. Chemical modifications that enhance stability (such as LNA or 2′-O-methyl modifications) can provoke immunogenicity, necessitating a delicate balance between persistence and safety [277]. Fourth, pharmacokinetics and dosing remain difficult to optimize. There is limited understanding of how to maintain a therapeutic window over time without eliciting adverse effects, given the long tissue half-life of modified oligonucleotides and their potential accumulation [120]. Fifth, manufacturing and regulatory obstacles pose significant challenges. It is difficult to produce chemically modified miRNAs on a large scale with batch-to-batch consistency, and non-coding RNA therapies must adhere to strict safety, quality, and reproducibility standards [280]. It is still difficult to achieve GMP-grade, scalable production without sacrificing the chemical changes needed for stability and effectiveness [121]. Lastly, little is known about miRNA biology in disease settings, particularly in heart disease in humans. Preclinical results may not necessarily translate predictably in patients due to the context-dependent actions of miRNAs (e.g., cell-type specificity, dynamic regulation) [284]. To overcome these translational challenges, deeper mechanistic research is required to improve miRNA target selection and minimize pleiotropic effects [285]. Additionally, the development of immunocompatible oligonucleotide chemistries, robust translational pipelines incorporating scalable manufacturing and early regulatory engagement, and innovative delivery platforms, such as exosome-based systems that are intrinsically biocompatible and may enhance cardiac specificity, will be essential [286].

#### Limitations in RNA Clinical Application

Despite substantial advances in RNA biology and growing clinical interest, the translation of RNA-based approaches, including miRNA-based diagnostics and therapeutics, into routine cardiovascular practice remains constrained by several fundamental limitations. These challenges span biological complexity, technical variability, clinical validation, and regulatory implementation, and collectively explain why RNA-based tools have not yet achieved widespread clinical adoption in heart failure management [101,102,103]. A major biological limitation arises from the context-dependent and pleiotropic nature of miRNA function. Individual miRNAs often regulate hundreds of target transcripts across multiple cell types, and their net biological effect depends on disease stage, tissue specificity, and the surrounding molecular environment [54,68,100]. In heart failure, where cardiomyocytes, fibroblasts, endothelial cells, and immune cells contribute dynamically to disease progression, this complexity complicates both mechanistic interpretation and therapeutic targeting [22,104]. Consequently, modulation of a single miRNA may produce beneficial effects in one cellular compartment while eliciting unintended consequences in others, raising concerns regarding safety and predictability [180,287]. From an analytical perspective, substantial pre-analytical and methodological variability limits reproducibility across studies. Differences in biospecimen type (serum vs. plasma vs. whole blood), RNA isolation protocols, haemolysis control, detection platforms (qPCR, microarrays, next-generation sequencing), and normalization strategies lead to inconsistent quantification of circulating RNAs [191,229,267]. These discrepancies impede direct comparison between cohorts and undermine the establishment of universally accepted diagnostic thresholds or reference ranges [252,268]. Although recent efforts toward standardization and population-based reference intervals are promising [252,266], harmonized workflows remain essential before RNA biomarkers can be reliably implemented in clinical laboratories. Clinical validation represents an additional bottleneck. Many RNA biomarker studies in heart failure are limited by small sample sizes, single-center designs, and enrichment for specific phenotypes, most commonly HFrEF, restricting generalizability [103,106]. Sex-specific effects, ethnic variability, comorbidities such as chronic kidney disease or diabetes, and treatment-related confounders are often insufficiently addressed [105,288]. Furthermore, while numerous RNA signatures demonstrate statistical associations with disease severity or outcomes [130,254,289], fewer studies have rigorously tested their incremental value beyond established clinical scores, natriuretic peptides, and imaging markers in prospective, adequately powered trials [107,108,109]. Therapeutic application of RNA-based strategies faces additional hurdles. Efficient, cardiac-specific delivery remains a central challenge, as systemically administered RNA molecules preferentially accumulate in the liver and kidneys [121,122,173]. Chemical modifications that enhance stability and cellular uptake may simultaneously increase immunogenicity or off-target effects [277,279]. Moreover, the long tissue half-life of modified oligonucleotides complicates dose titration and raises concerns regarding reversibility in the event of adverse effects [102,169]. These issues necessitate careful pharmacokinetic and pharmacodynamic characterization, which remains limited for most RNA-based cardiovascular therapies [120,287]. Finally, regulatory and translational barriers further delay clinical implementation. RNA-based diagnostics and therapeutics require stringent quality control, batch-to-batch consistency, and long-term safety evaluation [121,280]. The absence of well-defined regulatory pathways for complex RNA panels, combined with high manufacturing costs and scalability challenges, currently restricts their deployment outside specialized research settings [102,279]. In summary, although RNA-based approaches offer unique mechanistic insight and substantial potential for precision medicine in heart failure, their clinical application is constrained by biological complexity, technical variability, limited large-scale validation, delivery challenges, and regulatory uncertainty. Addressing these limitations through standardized methodologies, robust multicenter studies, and continued innovation in delivery and regulatory science will be essential to unlock the full clinical potential of RNA-based diagnostics and therapeutics [101,102].

### 7.3. Preclinical and Early Clinical Evidence

The pathological course of heart failure (HF) can be significantly influenced by the manipulation of microRNAs (miRNAs), particularly those with profibrotic and pro-remodeling functions, including miR-21, miR-34a, miR-133a, miR-132, and miR-208a, highlighting their wide translational potential [290]. miR-21 remains a central target for antifibrotic therapy. In a clinically relevant porcine ischemia/reperfusion model, intracoronary administration of a locked-nucleic-acid (LNA) antimiR-21 significantly reduced interstitial fibrosis and cardiomyocyte hypertrophy and improved cardiac function over 33 days. Transcriptomic analysis demonstrated suppression of MAPK and inflammatory signaling, while cellular deconvolution identified reductions in fibroblast and macrophage populations [144]. Mechanistically, miR-21 promotes fibroblast proliferation via SPRY1 inhibition, unleashing ERK/MAPK signaling, and in HFpEF models, overexpression increases Bcl-2, enhancing fibroblast survival and extracellular matrix accumulation [287]. Recent ex vivo studies in human failing myocardium confirmed that LNA-antimiR-21 reduces pro-fibrotic gene expression, suggesting translational relevance to human HF [291]. Collectively, these findings validate miR-21 as a viable target for large-animal and potentially clinical HF interventions [102]. miR-34a has also been extensively studied. In a mouse model of diabetes-induced cardiomyopathy, LNA-based inhibition modestly limited cardiac enlargement and fibrosis, although diastolic function improvements were limited [292]. Small-molecule modulation, such as dihydromyricetin (DHM) treatment, downregulated miR-34a, restored autophagy, reduced apoptosis, and ameliorated cardiac dysfunction in diabetic mice [293]. Mechanistically, miR-34a directly binds to the 3′ UTR of Smad4, a key mediator of TGF-β signaling in fibroblasts, promoting collagen synthesis and fibrosis [292,294]. Comprehensive reviews further highlight its dual role in regulating cell cycle, apoptosis, and fibrosis, emphasizing the need for carefully targeted approaches [198]. miR-133a has multifaceted benefits in diabetic cardiomyopathy. Systemic or cardiac-specific overexpression limits fibrosis, improves contractility, normalizes β-adrenergic signaling, and reduces activation of profibrotic pathways under chronic metabolic stress [288]. Additionally, miR-133a appears to protect against diabetes-induced lipotoxicity in the myocardium, suggesting metabolic-stabilizing effects beyond structural remodeling [147]. miR-132 inhibition has progressed to large-animal models, providing translational evidence for its therapeutic potential. In a porcine model of pressure-overload HF induced by percutaneous aortic constriction, intracoronary administration of a LNA-based antisense inhibitor of miR-132 (CDR132L) attenuated cardiomyocyte hypertrophy, reduced interstitial fibrosis, preserved myocardial compliance, and maintained capillary density [295]. Functionally, these improvements translated into enhanced global cardiac performance, including increased ejection fraction, improved stroke volume, and favorable left-ventricular strain parameters. Mechanistically, miR-132 inhibition likely acts through de-repression of FOXO3, promoting autophagy and counteracting maladaptive hypertrophic signaling, while also modulating profibrotic TGF-β/Smad3 pathways [281]. Collectively, these findings demonstrate that inhibiting multiple miRNAs, such as miR-21, miR-34a, miR-133a, and miR-132, can simultaneously reduce fibrosis, hypertrophy, metabolic stress, and microvascular rarefaction, supporting their potential as disease-modifying therapies in HF [86]. However, translation to clinical use requires careful optimization, including long-term safety, immunogenicity, off-target effects, and tissue-selective delivery methods [277].

### 7.4. Integration with Multi-Omics and Systems Biology

Integrating microRNA (miRNA) profiling with multi-omics technologies, including proteomics, metabolomics, transcriptomics, and epigenomics, represents a central strategy for the development of miRNA-based therapies in heart failure (HF). Multi-omics approaches enable the characterization of complex molecular networks that single-layer analyses cannot capture, thereby uncovering regulatory interactions relevant to HF pathogenesis [128]. By aligning miRNA expression profiles with quantitative protein abundance and metabolite fluxes, multi-omics frameworks provide a systems-level understanding of how miRNAs contribute to fibrosis, metabolic remodeling, inflammation, and impaired contractility. This integrated perspective facilitates the discovery of therapeutic miRNAs, enables improved disease stratification, and reveals regulatory nodes amenable to pharmacological intervention. The utility of this approach has been demonstrated in atrial-fibrillation-associated HF, where proteomic–metabolomic integration revealed dysregulated pathways with potential miRNA regulators [296]. Large-scale human multi-omics datasets further underscore the potential for cross-referencing miRNA–target interactions. Providencia et al. identified 35 metabolites and 38 druggable cardiac-expressed proteins associated with HF and atrial fibrillation via Mendelian randomization, producing a molecular atlas that can be readily mapped to miRNA–target databases [297]. Similarly, proteomic–metabolomic analyses in HFpEF cohorts revealed coordinated shifts in immune and inflammatory pathways, suggesting upstream miRNAs that may regulate these perturbations [298]. Such datasets form the foundation for systems-biology models capable of prioritizing candidate miRNAs according to their connectivity to disease-relevant molecular modules. Network-based multi-omics approaches allow reconstruction of regulatory pathways by integrating miRNA–mRNA interactions with differentially expressed proteins and metabolic signatures [129]. Overlaying miRNA profiles onto metabolite–protein interaction networks help distinguish central pathological regulators from marginally relevant miRNAs. This principle is further supported by whole-heart multi-omics studies in murine HF models, where core miRNAs linked to metabolic dysregulation and impaired energy homeostasis were identified [299]. A systems-level perspective is essential for narrowing therapeutic miRNA candidates to those influencing metabolic, structural, and inflammatory modules. This view aligns with broader analyses of non-coding RNA therapeutics, highlighting the potential of targeting highly connected miRNA hubs [102]. The power of integrated miRNA–omics approaches is further illustrated in human fetal heart development, where combined small-RNA, mRNA, and proteomic analyses revealed that miR-133a-3p regulates metabolic maturation, demonstrating the translational relevance of linking miRNA shifts to coherent transcriptomic and proteomic remodeling [300]. Epigenetic mechanisms add another regulatory dimension. Multi-omics analyses in pressure-overload HF models show that differentially expressed miRNAs, such as let-7b-5p and miR-23b-3p, modulate m^6^A RNA-modifying enzymes, including FTO and IGF2BP2, reshaping the epitranscriptomic landscape in the failing heart [301]. Supporting evidence for METTL3-dependent m^6^A regulation in cardiac remodeling is rapidly accumulating, with recent work indicating increased m^6^A modification and METTL3 upregulation in pressure-overload and ischemic models [302]. These studies reinforce miRNA–epitranscriptomic interactions in HF. By linking changes in miRNA expression to both downstream proteometabolic changes and epitranscriptomic enzymes, the integrated multi-omics approach supports these mechanistic discoveries. Targeted perturbations (knockout or inhibition) of METTL3 or related m6A readers/writers can mitigate pathological remodeling in vivo. For instance, TAC and ischemia/reperfusion studies have shown induction of METTL3 and global m6A increases associated with altered miRNA profiles [303]. Systems-biology and network-level modeling are essential for identifying miRNA regulatory hubs with multi-layer influence. Computational approaches, including machine-learning feature selection and multi-omics integration, have effectively identified candidate miRNA regulators in human and animal HF cohorts [304]. Integrated transcriptomic–proteomic analysis of ischemic HF identified pleiotrophin (PTN) as a key immune–inflammatory mediator and predicted upstream miRNAs modulating PTN-driven pathways [305]. Other systems analyses confirm that miRNAs such as miR-21-5p and miR-199a-5p regulate mitochondrial, cytoskeletal, and fibrotic modules, supporting their roles as multi-pathway intervention points [306]. The identification of miRNAs involved in fibroblast–macrophage communication and other intercellular circuits pertinent to HFpEF and HFrEF has been further refined by cell-type-resolved multi-omics, which includes single-cell or single-nucleus transcriptomics combined with circulating small-RNA and tissue proteomics [307]. Multi-omics integration still has analytical difficulties despite these developments. High-dimensional datasets require thorough preprocessing, including imputation, normalization, and batch correction, in addition to interpretable AI-based methods since they contain noise, batch effects, and missing values [308]. Recent advances in AI-based missing-data imputation using generative models and statistical learning have improved handling of sparsity in multi-omics datasets [309]. Single-cell multi-omics integration frameworks, such as scMaui, effectively address batch effects and missing modalities while preserving biological signal [310]. Biological heterogeneity across human cohorts, animal models, and in vitro systems complicates causal inference, so predicted miRNA–target interactions require experimental validation using CRISPR perturbation, antimiR or mimic studies, and orthogonal proteomic or metabolic assays [311]. Clinical translation of miRNA therapies is limited by challenges in delivery, including tissue specificity, biodistribution, immunogenicity, and chronic safety. Effective carriers must prevent immune activation, achieve target-tissue uptake, and protect miRNAs from degradation [122]. Multi-omics-guided prioritization provides a powerful platform for identifying therapeutically relevant miRNA candidates, but successful translation also depends on advances in delivery technologies.

### 7.5. Artificial Intelligence and Personalized Medicine

Artificial intelligence (AI) applied to miRNA datasets enables network analyses, patient stratification, and prediction of therapy responses. Machine-learning models built on high-dimensional miRNA expression data can categorize patient subgroups with distinct prognoses or likelihoods of responding to miRNA-based therapies, such as in cancer or cardiovascular disease [312]. AI approaches also identify miRNA signatures that highlight key regulatory miRNAs for treatment while differentiating between disease states, integrating multi-omics data such as transcriptomics and proteomics to predict prognostic miRNAs and mRNA targets [313]. Patient-specific miRNA profiles combined with clinical and molecular data can optimize miRNA-based treatments (mimics or inhibitors), maximize therapeutic benefit while minimize side effects [314]. Algorithmic prioritization with precision delivery methods, such as exosomes or nanoparticles, further enhances the feasibility of individualized therapy [315]. Advanced bioinformatics pipelines integrating target prediction, network modeling, generative AI models, and NGS miRNA data support translational applications in precision oncology [316]. In heart failure (HF), AI-driven analysis of patient-specific miRNA profiles, integrated with clinical variables, can reveal regulatory signatures stratifying patients by prognosis or therapy response likelihood [317]. Circulating miRNAs identified through next-generation sequencing in advanced HF patients serve as predictive biomarkers, and multivariate models using these miRNAs improve risk stratification beyond conventional clinical indicators [318]. Integration of miRNA and transcription factor (TF) networks has identified key regulatory molecules involved in HF pathogenesis, offering mechanistic insights for therapy design. Regulatory network analysis based on integrated miRNA-TF reveals key genes in heart failure [319]. Machine-learning approaches have also been applied to HFpEF. Circulating miRNA profiles analyzed using LASSO regression, random forest, and other algorithms have identified small panels of miRNAs (e.g., hsa-miR-199b-5p, hsa-miR-296-5p) capable of accurately distinguishing HFpEF patients from controls [320]. These AI-derived signatures can serve as therapeutic targets or biomarkers in personalized medicine frameworks, supporting interventions that rebalance beneficial versus harmful miRNAs in specific patients [318]. Combining multi-omics data, including miRNA, mRNA, and TF networks, with AI facilitates hypothesis generation for combination therapies, with systems biology studies identifying miRNA signatures predictive of cardiac remodeling [321]. Despite these advances, challenges remain. AI models require large, well-annotated cohorts, and meta-analyses highlight the need for standardization and validation of miRNA profiles across diverse populations [322]. Therapeutic translation also depends on safe and effective in vivo delivery of prioritized miRNAs, as highlighted in recent multi-analyte liquid biopsy studies in HF [219].

### 7.6. Early Detection and Preventive Potential

Circulating microRNAs (c-miRNAs) are emerging as powerful non-invasive biomarkers for the early detection of heart failure (HF). By capturing molecular changes that precede overt cardiac remodeling, c-miRNAs can identify individuals at risk before clinical symptoms become apparent, complementing imaging and functional evaluations [219,289]. Recent studies have validated novel miRNAs for early HF detection. For instance, miR-320a-3p has been associated with HF diagnosis and adverse outcomes in chronic HF patients, while miR-34a is upregulated in early disease stages, indicating its potential as an early biomarker [323]. Additional work has established reference intervals for circulating miRNAs such as miR-1-3p, miR-23a-3p, and miR-21-5p in healthy populations, which is critical for distinguishing pathological elevation from normal variation [252]. Systematic reviews and meta-analyses emphasize the diagnostic and prognostic value of multi-miRNA panels in HF, showing strong predictive accuracy for progression to symptomatic disease across diverse cohorts [271]. These findings support the potential for miRNA-guided preventive strategies, such as targeted miRNA mimics or inhibitors tailored to an individual’s molecular profile, aimed at delaying or preventing pathological remodeling. Precision prevention in HF may be achievable by combining c-miRNA signatures with clinical, imaging, and functional data to identify high-risk individuals early, allowing for tailored interventions to preserve cardiac function and slow disease progression [324].

## 8. Conclusions

MicroRNAs are central regulators of cardiac remodeling and are strongly linked to the development and progression of heart failure. Although many miRNAs, including miR-21, miR-34, miR-155, miR-320a-3p, and miR-199b-5p, show promise as biomarkers and therapeutic targets, key questions remain. It is still unclear which miRNA signatures are robust and reproducible across diverse populations, how early circulating miRNAs truly reflect disease mechanisms, and which networks drive specific heart failure phenotypes. Large, well-defined patient cohorts and standardized protocols for sampling and analysis are urgently needed to validate candidate miRNAs and define reliable reference ranges, including for miR-1-3p, miR-23a-3p, and miR-21-5p. Artificial intelligence and multi-omics integration offer powerful tools to identify clinically meaningful miRNA patterns, but these models require prospective validation and transparent performance testing before clinical adoption. Mechanistic studies must also move beyond association to clarify how specific miRNA networks interact with transcription factors, mRNAs, and signaling pathways in different stages of heart failure.

For therapy, the main challenges are safe and targeted delivery, long-term safety, and avoidance of off-target effects. Nanoparticle- and exosome-based systems are promising but need rigorous evaluation in large preclinical and clinical studies. Future research should prioritize defining disease-specific miRNA networks, standardizing circulating miRNA measurement, validating AI-based prediction models, and developing safe delivery platforms. Future studies should prioritize longitudinal designs, phenotype-specific cohorts, and integrative multi-omic approaches capable of distinguishing causal regulatory networks from epiphenomenal signals. Addressing these priorities will determine whether miRNA-guided strategies can truly enable earlier detection, precise risk stratification, and personalized treatment in heart failure.

## Figures and Tables

**Table 1:** Representative archetypes of translational discordance between preclinical and human miRNA studies in heart failure.

miRNA	Mouse/Preclinical Models	Human Clinical Data	Translational Conflict	References
miR-21	Inhibition reduces fibrosis and improves remodeling in pressure-overload and MI models	Elevated circulating levels correlate with fibrosis severity and worse outcomes	Therapeutic inhibition beneficial in mice but elevated levels in humans reflect advanced disease rather than direct causality	[115,116,117,118]
miR-22	Overexpression promotes hypertrophy in mouse cardiomyocytes	Variable or modest associations in human HF cohorts	Strong hypertrophic effects in mice not consistently replicated clinically	[42,58]
miR-26a	Regulates fibroblast activation and ECM turnover in mouse models	Limited and heterogeneous clinical associations	Species-dependent effects on fibrosis pathways	[42,58,119]

**Table 2:** Clinical Cohort Evidence Linking Circulating microRNAs to Heart Failure Phenotype, Severity, and Outcomes.

miRNA	Cohort (n, Type)	HF Phenotype/Clinical Setting	Sample/Platform	Adjustment	Effect Size/Discrimination	Endpoint (Follow-Up)	Key Limitation Affecting Reproducibility	References
miR-423-5p	Moderate–large prospective and retrospective cohorts	HFrEF, mixed HF; predominantly chronic	Plasma/serum; qPCR	Multivariable in several cohorts	Modest diagnostic and prognostic signal (AUC~0.70–0.80 in some studies)	HF progression, mortality (mid-term follow-up)	Platform variability; incomplete reporting of normalization and hemolysis control in early studies	[86,252,255]
miR-21	Observational cohorts; variable size	HFrEF, HFpEF	Plasma/serum; qPCR	Often adjusted for clinical covariates	Associations with fibrosis and adverse outcomes; effect sizes heterogeneous	Mortality and remodeling progression	Limited tissue–blood concordance; residual confounding from comorbidities (renal dysfunction, metabolic disease)	[115,117,118,243]
miR-122	Cohort studies and meta-analysis	Mixed HF; cardiometabolic enrichment	Plasma; qPCR/sequencing	Adjusted in larger studies	Prognostic signal for cardiovascular death; modest incremental value	Cardiovascular mortality	Likely reflects systemic metabolic injury rather than cardiac-specific biology	[130,238,254]
miR-146a	Small–moderate observational cohorts	HFpEF	Plasma; qPCR	Limited multivariable adjustment	Phenotype discrimination more consistent than outcome prediction	HFpEF identification; limited longitudinal data	Small cohorts and limited external validation	[58,86,105]
miR-126	Population-based and HF cohorts	Mixed HF; vascular phenotype	Plasma; qPCR	Adjusted in population studies	Associated with incident HF and reduced functional capacity	Incident HF; exercise tolerance	Susceptible to pre-analytical variation due to platelet contamination	[252,256,257]
miR-210	Acute and chronic HF cohorts; generally moderate size	Advanced or hypoxic HF states	Plasma/serum; qPCR	Variable	Correlates with disease severity; prognostic value emerging	Severe HF outcomes	Context-sensitive expression (hypoxia-driven); limited longitudinal validation	[92,248]

**Table 3:** Therapeutic Targeting of miRNAs in Heart Failure: Delivery Strategies, Clinical Progress, and Translational Barriers.

miRNA Target	Therapeutic Modality/Chemistry	Delivery (Vehicle, Route, Dosing)	Development Stage	Primary Therapeutic Signal	Safety/Off-Target Considerations	Outcome or Failure Driver	References
miR-132	LNA-modified antimiR (CDR132L)	Systemic delivery; intravenous administration in clinical studies; repeat dosing evaluated	Large-animal models; Phase 1b completed; Phase II ongoing	Reduced cardiac hypertrophy and fibrosis; improved LV function and remodeling	Favorable early safety profile; no major dose-limiting toxicity reported	Most clinically advanced candidate; long-term efficacy and scalability remain under evaluation	[110,111,112,113,114,275,276]
miR-21	AntimiR/antisense inhibition	Primarily systemic delivery in murine models	Preclinical	Attenuation of fibrosis and adverse remodeling	Broad target network raises risk of pleiotropic effects	Inconsistent reproducibility across models; uncertain human therapeutic window	[116,117]
miR-34a	Antisense inhibition	Systemic administration in rodents	Preclinical	Reduced apoptosis and fibrotic signaling	Potential off-target gene regulation; limited cardiac specificity	Translational uncertainty due to insufficient large-animal and human data	[58,123]
Multiple miRNAs	Mimics and inhibitors (various platforms)	Lipid nanoparticles, viral vectors, and conjugated oligonucleotides explored	Early preclinical	Modulation of remodeling pathways	Immunogenicity, hepatic accumulation, and unintended gene-network effects	Delivery efficiency and tissue specificity remain the dominant barriers to clinical translation	[120,121,277,278,279,280]

## Data Availability

No new data were created or analyzed in this study. Data sharing is not applicable to this article.

## Abbreviations

AGO	Argonaute protein
AGO2	Argonaute 2
AI	Artificial intelligence
AKT	Protein kinase B
Ang II	Angiotensin II
ASK1	Apoptosis signal-regulating kinase 1
AT1	Angiotensin II type 1 receptor
ATP	Adenosine triphosphate
β-MHC	Beta-myosin heavy chain
BCL2	B-cell lymphoma 2
BIM	BCL2-interacting mediator of cell death
BNP	B-type natriuretic peptide
CaMKII	Ca^2+^/calmodulin-dependent protein kinase II
CCR4–NOT	Carbon catabolite repression 4 negative on TATA-less
CDR132L	LNA-based anti-miR-132
ceRNA	Competing endogenous RNA
CMR	Cardiac magnetic resonance
COL1A1	Collagen type I alpha 1
COL3A1	Collagen type III alpha 1
CTGF	Connective tissue growth factor
CPT1A	Carnitine palmitoyltransferase 1A
DCM	Dilated cardiomyopathy
DGCR8	DiGeorge syndrome critical region 8
DHM	Dihydromyricetin
DNA	Deoxyribonucleic acid
ECM	Extracellular matrix
ECV	Extracellular volume
EF	Ejection fraction
EndoMT	Endothelial-to-mesenchymal transition
ERK	Extracellular signal-regulated kinase
FAK	Focal adhesion kinase
FAO	Fatty acid oxidation
FOXO3	Forkhead box O3
FTO	Fat mass and obesity-associated protein
Gal-3	Galectin 3
GATA4	GATA binding protein 4
GPX4	Glutathione peroxidase 4
GW182	Trinucleotide repeat-containing protein 6
TNRC6	Trinucleotide repeat-containing protein 6
HDAC	Histone deacetylase
HDL	High-density lipoprotein
HF	Heart failure
HFpEF	Heart failure with preserved ejection fraction
HFrEF	Heart failure with reduced ejection fraction
HMC	Hypertrophic cardiomyopathy
IGF-1	Insulin-like growth factor 1
IGF-1R	Insulin-like growth factor 1 receptor
IGF2BP2	Insulin-like growth factor 2 mRNA-binding protein 2
IL	Interleukin
Jpx	Long non-coding RNA Jpx
LA	Left atrium
LASSO	Least absolute shrinkage and selection operator
LNA	Locked nucleic acid
lncRNA	Long non-coding RNA
LV	Left ventricle
MAPK	Mitogen-activated protein kinase
MEF2	Myocyte enhancer factor 2
METTL3	Methyltransferase-like 3
MI	Myocardial infarction
miRNA	MicroRNA
miR	MicroRNA
m6A	N6-methyladenosine
MMP	Matrix metalloproteinase
MSC	Mesenchymal stem cell
NFAT	Nuclear factor of activated T cells
NF-κB	Nuclear factor kappa B
NLRP3	NOD-like receptor family pyrin domain-containing 3
NPM1	Nucleophosmin 1
NT-proBNP	N-terminal pro-B-type natriuretic peptide
NYHA	New York Heart Association
PCD	Programmed cell death
PCR	Polymerase chain reaction
PDLIM5	PDZ and LIM domain protein 5
PI3K	Phosphoinositide 3-kinase
PINK1 PTEN	induced kinase 1
PTEN	Phosphatase and tensin homolog
RAAS	Renin–angiotensin–aldosterone system
RISC	RNA-induced silencing complex
RNA	Ribonucleic acid
ROS	Reactive oxygen species
SERCA2a	Sarcoplasmic reticulum Ca^2+^-ATPase
sST2	Soluble suppression of tumorigenicity 2
SGLT2i	Sodium–glucose co-transporter 2 inhibitor
SMAD	Mothers against decapentaplegic homolog
SORBS2	Sorbin and SH3 domain-containing protein 2
SRF	Serum response factor
TAC	Transverse aortic constriction
TGF-β	Transforming growth factor beta
TLR	Toll-like receptor
TMG	Trimethylguanosine
TNF-α	Tumor necrosis factor alpha
TRBP	TAR RNA-binding protein
UTR	Untranslated region
VEGF	Vascular endothelial growth factor
Wnt	Wingless-related integration site
XPO1	Exportin 1
YAP	Yes-associated protein
TAZ	Transcriptional co-activator with PDZ-binding motif
